# Modeling an SPR Sensor for Carcinoma-Related Refractive-Index Detection: The Case of CaF_2_/Au/Si_3_N_4_/BP Multilayer System

**DOI:** 10.3390/bios16040198

**Published:** 2026-04-01

**Authors:** Talia Tene, Martha Ximena Dávalos Villegas, Cristian Vacacela Gomez

**Affiliations:** 1 Department of Chemistry, Universidad Técnica Particular de Loja, Loja 110160, Ecuador; 2 Facultad de Ciencias, Escuela Superior Politécnica de Chimborazo (ESPOCH), Panamericana Sur km. 1 1/2, Riobamba 060155, Ecuador; 3 Department of Physics, University of Calabria, Via P. Bucci, Cubo 33C, 87036 Rende, Italy; 4 Universidad Ecotec, Km. 13.5 Samborondón, Samborondón EC092302, Ecuador

**Keywords:** surface plasmon resonance, optical nanosensor, black phosphorus, carcinoma-related sensing, prism-coupled biosensor, transfer-matrix method, refractive-index sensing

## Abstract

A thin-film surface plasmon resonance (SPR) sensor is presented using a prism-coupled Kretschmann configuration and an optimized multilayer architecture incorporating black phosphorus (BP) as an ultrathin overlayer. The response is modeled at 633 nm under TM polarization using the transfer-matrix method. Low-concentration sensing conditions in the 1–5 ng/mL range are represented through small effective-refractive-index perturbations of the aqueous sensing medium, providing a preliminary optical framework for evaluating refractive-index response in biosensing-related scenarios. The coupling prism, Au film thickness, and Si_3_N_4_ spacer thickness are optimized to control resonance depth, linewidth, and angular shift. The optimized CaF_2_/Au/Si_3_N_4_/BP configuration exhibits systematic condition-dependent displacement of the SPR minimum and an evanescent-field distribution that remains strongly localized at the sensing interface while extending into the sensing medium, enabling refractive-index interrogation. High angular sensitivity is obtained at low levels, reaching 517.62°/RIU at 2 ng/mL and 482.82°/RIU at 1 ng/mL, with quality factors above 120 RIU^−1^ in the same regime. Composite indicators (figure of merit and contrast signal factor) peak at intermediate levels, whereas resonance broadening at higher levels reduces the quality factor and increases the inferred limit of detection, evidencing a sensitivity–resolution trade-off. Benchmarking against reported SPR platforms indicates that BP-assisted interface engineering provides a competitive low-level operating window within a preliminary refractive-index-sensing framework that is relevant to future biosensor design. These results motivate further experimental validation, including BP stabilization, surface biofunctionalization, and practical implementation under liquid-phase sensing conditions.

## 1. Introduction

Carcinoma remains a major cause of morbidity and mortality worldwide, and clinical outcomes depend strongly on the stage at diagnosis [1,2]. Earlier detection increases the range of therapeutic options and supports longitudinal monitoring during and after treatment [3]. In this context, optical biosensing has attracted interest for detecting low-level biomarker-associated changes in complex biofluids, such as serum, plasma, and saliva, where analytical performance depends on sensitivity, specificity, and measurement reproducibility [4]. Among label-free optical methods, approaches that monitor refractive-index variations at a solid–liquid interface are especially relevant because they convert interfacial molecular interactions into real-time quantitative signals [5,6].

Thin-film optical biosensors are central to current diagnostic research because engineered multilayer stacks and ultrathin functional coatings can strengthen electromagnetic-field confinement near the sensing interface and improve the response to small dielectric perturbations [7,8,9,10]. In particular, prism-coupled SPR platforms provide a mature and reproducible route for label-free interfacial sensing, while functional overlayers, including two-dimensional materials, can modify field distribution and interfacial interaction conditions [10,11]. Hybrid SPR architectures combining plasmonic metals with dielectric, oxide, and two-dimensional functional layers, such as graphene derivatives, TMDCs, and ITO-based overlayers, have also been widely explored to tailor resonance position, field confinement, and sensing performance in multilayer sensor platforms [12,13,14]. These features make SPR an attractive framework for preliminary evaluation of material combinations that are intended for biosensing applications.

A further driver is the demand for non-invasive or minimally invasive testing. Compared with tissue biopsy, nanosensor-based assays can be designed to interrogate biofluids and support repeated measurements for screening and follow-up [15,16]. This is particularly relevant in liquid-biopsy-oriented settings, where clinically informative species may appear at low levels and require sensitive transduction schemes, although different carcinoma-related biomarkers span distinct physicochemical classes, concentration windows, and interfacial requirements [17]. Accordingly, the present study does not focus on a specific capture chemistry or a single analyte family but instead examines the optical response of the SPR platform under effective-refractive-index perturbations that are representative of low-concentration sensing conditions. In this form, the analysis emphasizes the optical response trends of the multilayer structure without explicitly resolving target-specific capture layers, interfacial adlayer formation, or transport-limited binding effects.

In plasmonic architectures, gold (Au) remains a practical plasmonic metal because of its chemical stability, established biocompatibility in aqueous and physiological environments, and well-defined surface chemistry [18,19]. Au supports stable plasmonic resonances and provides reliable functionalization routes, including thiol-based immobilization strategies, which are widely used in experimental biosensor development [20]. These characteristics make Au a robust platform for prism-coupled SPR configurations that are intended for future biointerface integration [21].

The dielectric and photonic stack also plays a decisive role in field distribution, propagation loss, and device integration. Silicon nitride (Si_3_N_4_) is relevant because it is mechanically stable, exhibits low optical loss over common photonic bands, and is compatible with CMOS-type fabrication, enabling scalable manufacturing and on-chip integration of optical transducers [22,23]. When combined with Au, Si_3_N_4_ can function as a waveguiding or intermediate layer that helps to engineer the evanescent field at the sensing interface and supports compact sensor layouts that are suitable for multiplexing [24,25].

Black phosphorus (BP) has also attracted attention as a two-dimensional material for biosensing-oriented photonic and plasmonic platforms because its high surface-to-volume ratio and tunable optoelectronic behavior can strengthen interfacial light–matter interaction [26,27]. In hybrid Au-based SPR structures, BP can therefore be considered as an ultrathin overlayer of theoretical interest because its optical and dielectric characteristics may influence field confinement and the interfacial response of the multilayer platform. However, its practical use requires caution because BP is susceptible to degradation in ambient and aqueous environments. In the present work, BP is treated as an idealized ultrathin overlayer within the numerical framework, whereas experimental implementation would require stabilization, passivation, or biofunctionalization strategies. Such protective or functional interface layers would modify the outer optical stack and could shift the resonance condition relative to the idealized configuration analyzed here.

Within this framework, the combination of Au for stable plasmonic transduction, Si_3_N_4_ for dielectric-field control and photonic compatibility, and BP as an ultrathin outer overlayer provides a useful material basis for the theoretical evaluation of multilayer SPR performance. In this work, we present a transfer matrix method (TMM)-based stepwise optimization of a prism-coupled Au/Si_3_N_4_/BP thin-film SPR sensor, including prism selection, plasmonic film tuning, dielectric-spacer evaluation, and nanomaterial-assisted interface engineering under angular interrogation at 633 nm. The study then examines the concentration-dependent optical response of the adopted CaF_2_/Au/Si_3_N_4_/BP configuration through effective-refractive-index perturbations in the 1–5 ng/mL range. The main objective is to identify the optical operating window of this multilayer platform and to clarify the trade-offs between angular sensitivity, resonance sharpness, and related performance metrics within a preliminary refractive-index-sensing framework that is relevant to future biosensing design.

## 2. Materials and Methods

This work evaluates multilayer surface plasmon resonance (SPR) biosensor architectures designed for optical detection of carcinoma-associated refractive index (RI) changes under ultralow-concentration conditions. The optical response of each configuration was modeled using the TMM (see Supplementary Materials [28,29]), which enables angle-resolved reflectance calculations for stratified media by enforcing continuity of the tangential electric- and magnetic-field components at each interface. The simulations were carried out at a fixed working wavelength of λ = 633 nm (He–Ne line) using TM-polarized illumination to satisfy the excitation condition for surface plasmons at the metal–dielectric boundary.

### 2.1. Performance Metrics and Optimization Protocol

The baseline and optimized sensors were defined as N-layer stacks composed of (i) a prism/substrate, (ii) an Au plasmonic film, and (iii) optional functional/intermediate layers followed by the sensing medium. The prism materials considered for angular interrogation were BK7, CaF_2_, CsF, and SF_6_, allowing systematic assessment of substrate RI on resonance position and angular shift. The sensing medium was modeled as either water (*n* = 1.3300) [30,31,32] or an effective-refractive-index condition (*n_eff_* = 1.3337, corresponding to the 1 ng/mL case) [33] used to represent a low-level optical perturbation in the sensing environment.

Optical constants and thicknesses were assigned from the literature values compiled in the Supplementary Materials, including Au with complex refractive index (0.1378 + 3.6196i) at 633 nm and a nominal thickness of 45 nm for the reference design [34]. Silicon nitride (Si_3_N_4_) was treated as a low-loss dielectric layer (n = 2.0394) [35], with thickness swept in the nanometer range, and black phosphorus (BP) was incorporated as an ultrathin functional layer (n = 3.5 + 0.01i; thickness 0.53 nm) [36] when evaluating 2D-material-enhanced architectures. These BP optical constants were adopted from the literature as an idealized reference for an ultrathin BP layer and do not explicitly distinguish between pristine, oxidized, encapsulated, or otherwise protected surface states.

### 2.2. Transfer-Matrix Modeling

The reflectance spectrum R(θ) was calculated using the TMM, a well-established formalism for optical analysis of multilayer media [37,38]. In this framework, the tangential electric- and magnetic-field components in the incident region are related to those in the last region of the multilayer stack through the global transfer matrix (Equation (S8)), whose elements are obtained from the ordered product of the characteristic matrices of all layers (Equation (S9)). Each layer is represented by a characteristic matrix (Equation (S10)) that depends on the corresponding phase term and auxiliary parameter (Equations (S11) and (S12)), defined by the layer thickness d_k_, dielectric constant ε_k_, the incidence angle θ, and the free-space wavelength λ_0_. After determining the global matrix elements, the Fresnel reflection coefficient and the corresponding reflectance R(θ) are computed as given in Equation (S13), yielding the SPR angular spectrum used for resonance-angle extraction and subsequent metric evaluation. The complete derivation and boundary-condition definitions are provided in the Supplementary Numerical Modeling section (Equations (S8)–(S13)), and the implementation was validated against a reference three-layer model (Figure S1) using data from the literature [39]. All simulations were performed under TM polarization using λ = 633 nm and an angular sampling of 5 × 10^4^ points.

### 2.3. Configurations Under Study

**Scheme 1:**
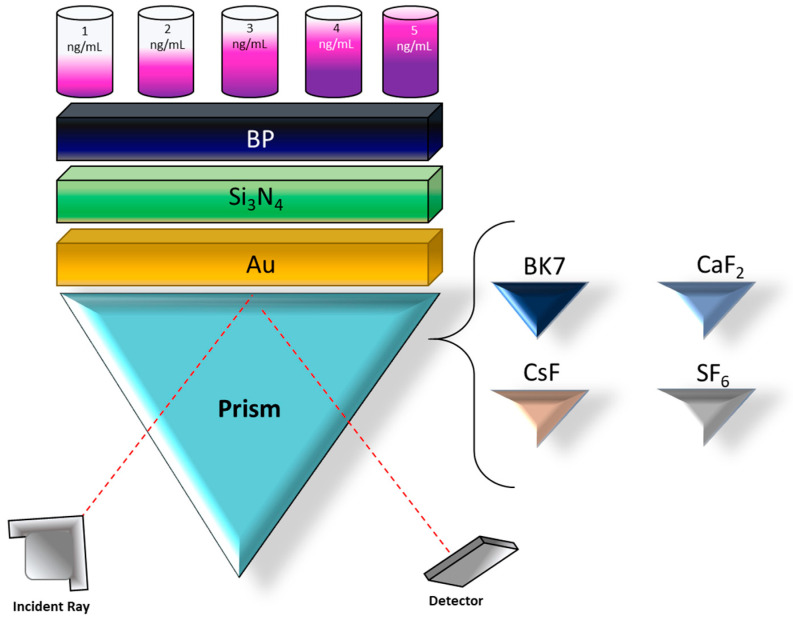
Kretschmann SPR thin-film sensor (prism/Au/Si_3_N_4_/BP) interrogated at 633 nm (TM) under modeled effective-refractive-index conditions associated with 1–5 ng/mL.

Scheme 1 summarizes the investigated Kretschmann SPR platform, where a coupling prism excites a surface plasmon supported by a thin Au film under TM-polarized illumination. To isolate the influence of (i) prism selection and (ii) functional overlayers on the optical response of the multilayer SPR platform under modeled low-concentration sensing conditions, multiple stacks were simulated under identical operating conditions using angular interrogation at a fixed wavelength of λ = 633 nm. This wavelength was selected as a standard SPR interrogation line (He–Ne), with widely tabulated optical constants for Au and common dielectrics and broad experimental accessibility in prism-coupled setups; wavelength-dependent optimization is left for future work. The sensing response was represented through small effective-refractive-index perturbations at the sensing interface within the numerical framework adopted here.

All the configurations share the common layout prism/Au/(optional dielectric layer)/(optional 2D layer)/sensing medium, as depicted in Scheme 1. The reference design contains Au as the sole plasmonic film. A dielectric-assisted variant introduces a Si_3_N_4_ interlayer on top of Au to tailor the evanescent-field distribution and stabilize the sensing interface. A 2D-enhanced variant further adds an ultrathin BP coating above Si_3_N_4_ to modify the optical response of the outer region of the multilayer structure under the assigned effective-RI conditions. The optical constants and nominal thicknesses used for the prism materials (BK7, CaF_2_, CsF, and SF_6_), Au, Si_3_N_4_, and BP are summarized in Table S2, together with the RI adopted for water and the effective-RI values used to define the modeled sensing conditions.

All the optical constants (including complex refractive indices where applicable) and nominal thicknesses for the prism materials (BK7, CaF_2_, CsF, and SF_6_), the Au film, the Si_3_N_4_ interlayer, the BP overlayer, and the sensing media at λ = 633 nm are detailed in Supplementary Table S2 together with the corresponding literature sources [29,30,31,32,33].

To quantify prism-driven effects on the resonance condition and the achievable angular shift, four coupling prisms were evaluated (BK7, CaF_2_, CsF, and SF_6_). For each prism, reflectance spectra R(θ) were computed for the baseline condition (water) and for the corresponding modeled effective-RI condition, and the resonance angle was extracted as the minimum of R(θ). The core comparison set used throughout the manuscript is listed in Table S1, where each tested system is uniquely defined by prism selection and the adopted sensing condition. Derived performance metrics (including sensitivity, full width at half maximum (FWHM), detection accuracy, figure of merit (FoM), QF, and limit of detection (LoD) were calculated from the resonance-angle shift between the baseline and modeled conditions and from the spectral characteristics of the SPR dip, ensuring that differences across systems originate from controlled changes in the multilayer stack rather than from illumination settings.

## 3. Results and Discussion

### 3.1. Prism Material Selection and Optimization

The prism material controls the in-plane wavevector of the incident field and therefore sets the phase-matching condition required to excite the surface plasmon at the Au interface. In the Kretschmann geometry (Scheme 1), changing the prism refractive index modifies both the resonance-angle position and the degree of field confinement at the metal–sensing-medium boundary. For this reason, prism selection is a first-order design step when optimizing an SPR sensor for ultralow refractive index (RI) perturbations under modeled low-concentration sensing conditions. For the prism screening in this section, the multilayer stack was kept fixed as a prism/Au/sensing medium at λ = 633 nm (TM), using the same Au optical constants and thicknesses (Table S2), so that only the prism material was varied.

**Figure 1:**
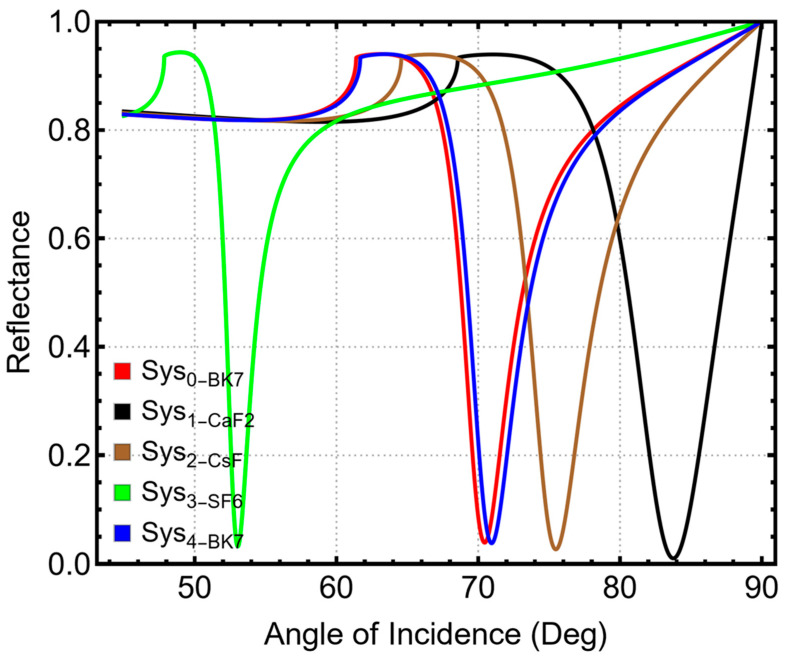
Prism-dependent SPR response at λ = 633 nm (TM): angular reflectance spectra R(θ) for different coupling prisms with an Au film, where the reflectance minimum defines the resonance angle θ_res_.

Figure 1 compares simulated angular reflectance spectra R(θ) at λ = 633 nm under TM polarization for prism-based systems using CaF_2_, CsF, SF_6_, and BK_7_. All the cases show a characteristic SPR minimum whose angular position depends on the prism material. As the prism RI increases, the resonance occurs at smaller incidence angles because the higher-index prism provides a larger in-plane wavevector component at a given angle, reducing the angle required to satisfy the plasmon coupling condition. This trend is quantified in Table S4: SF_6_ (n = 1.7990) yields the smallest resonance angle (53.04°), whereas CaF_2_ (n = 1.4329) shifts the resonance to a much higher angle (83.76°), with CsF and BK_7_ falling between these limits.

**Figure 2:**
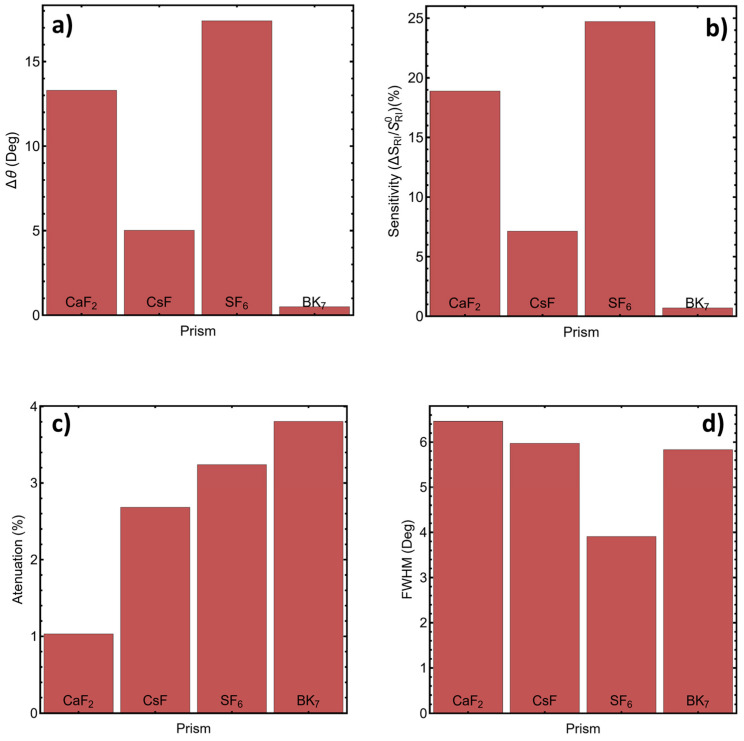
Prism-dependent SPR performance under the modeled 1 ng/mL sensing condition: (**a**) resonance-angle shift Δθ, (**b**) sensitivity enhancement relative to the reference configuration, (**c**) resonance attenuation, and (**d**) FWHM for the different coupling prisms (Table S3).

For sensing, the key quantity is the resonance-angle shift Δθ between the reference medium (water) and the modeled effective-refractive-index condition associated with 1 ng/mL because Δθ captures the transduction strength for a fixed perturbation. Figure 2a and Table S3 show that SF_6_ provides the largest angular shift (Δθ = 17.41°), followed by CaF_2_ (Δθ = 13.30°), while BK7 produces a negligible shift (0.49°). The same ranking appears in the sensitivity enhancement reported in Figure 2b and Table S3, where SF_6_ achieves 24.71% and CaF_2_ 18.88%, whereas BK7 reaches only 0.70%. These results indicate that, under identical wavelength and stack conditions, higher-index prisms yield a stronger mapping between small effective-RI perturbations and measurable resonance shifts.

The dip depth and linewidth provide complementary information about coupling strength and angular resolution. Figure 2c and Table S3 show that the resonance attenuation increases from CaF_2_ (1.03%) to BK7 (3.80%), meaning BK7 exhibits the deepest SPR minimum among the tested prisms despite having the smallest Δθ. This highlights that a deeper dip does not necessarily imply higher RI sensitivity; sensitivity is dominated by how strongly the resonance angle responds to Δn, whereas attenuation reflects the balance of coupling and losses in the optical stack. Linewidth is particularly important for angle interrogation because a narrower SPR dip generally improves the precision of resonance tracking. Figure 2d indicates that SF_6_ yields the smallest FWHM (3.90°), while CaF_2_ produces a broader resonance (6.46°). Combining Δθ and FWHM, SF_6_ shows the most favorable response within this theoretical prism set, illustrating the dependence of coupling on prism refractive index.

Overall, the prism comparison shows that increasing the prism refractive index strengthens the angular response and can also improve the linewidth under the same optical stack. Within this set, SF_6_ provides the strongest theoretical response and is therefore used as a high-index benchmark to illustrate the prism-index dependence of the coupling condition. For the subsequent multilayer optimization, however, CaF_2_ is selected as the working prism because it combines strong optical performance with greater practical relevance and experimental feasibility. This distinction between theoretical benchmarking and practical material selection is important for interpreting the design pathway adopted in the following sections.

### 3.2. Optimization of the Plasmonic Metal Layer

After establishing the prism-dependent coupling behavior, the thickness of the Au film was optimized because it directly controls plasmon excitation efficiency, radiative damping, and the shape of the SPR dip. In the Kretschmann configuration, Au thickness determines how effectively the evanescent field penetrates through the metal to reach the Au–sensing-medium interface. If the film is too thin, the mode can be overly lossy and the resonance becomes broadened; if it is too thick, the field transmission to the sensing interface is suppressed and the resonance becomes less responsive to small refractive index (RI) variations. The systems evaluated in this subsection are summarized in Table S5, where the CaF_2_ prism is kept fixed while the Au thickness is varied from 30 to 45 nm under identical angular interrogation at λ = 633 nm (TM polarization).

**Figure 3:**
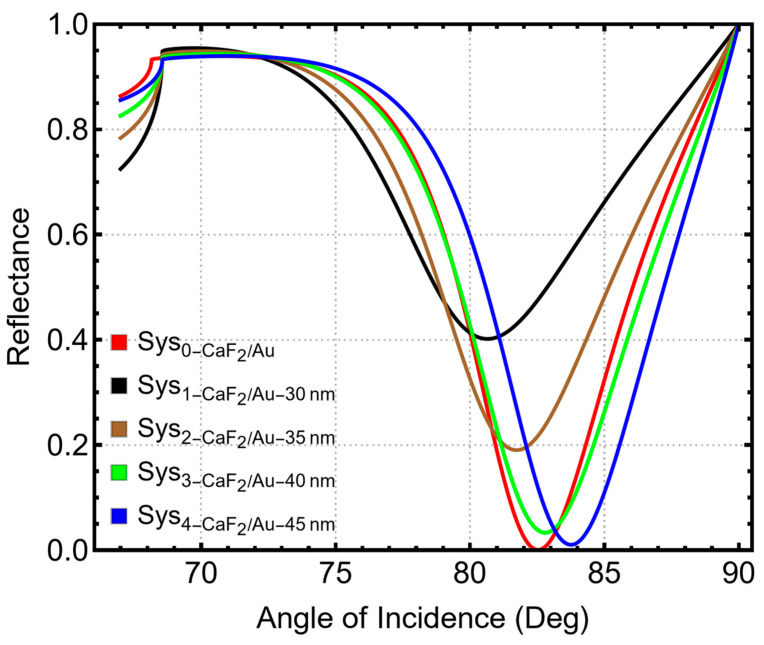
Angular reflectance spectra R(θ) for CaF_2_-coupled SPR at λ = 633 nm (TM) for Au thicknesses of 30–45 nm; the corresponding resonance angles are reported in Table S6.

Figure 3 shows the simulated angular reflectance spectra R(θ) for the CaF_2_-coupled SPR stack as a function of Au thickness. Increasing the Au thickness systematically shifts the resonance to higher incidence angles and modifies both the dip depth and linewidth. The extracted resonance positions reported in Table S6 increase from 80.64° (30 nm) to 83.75° (45 nm), consistent with the thickness-dependent dispersion of the coupled plasmonic mode. The most relevant observation for sensing, however, is that the resonance-angle shift between water and the modeled low-concentration sensing condition decreases markedly as the Au layer becomes thicker. Table S6 shows that Δθ drops from 0.40° at 30 nm to 0.19° at 35 nm and then becomes very small for 40 nm (0.03°) and 45 nm (0.01°). This behavior indicates that thicker Au films reduce the effective interaction between the evanescent plasmon field and the outer sensing region, which weakens the transduction of RI changes into measurable angular shifts.

**Figure 4:**
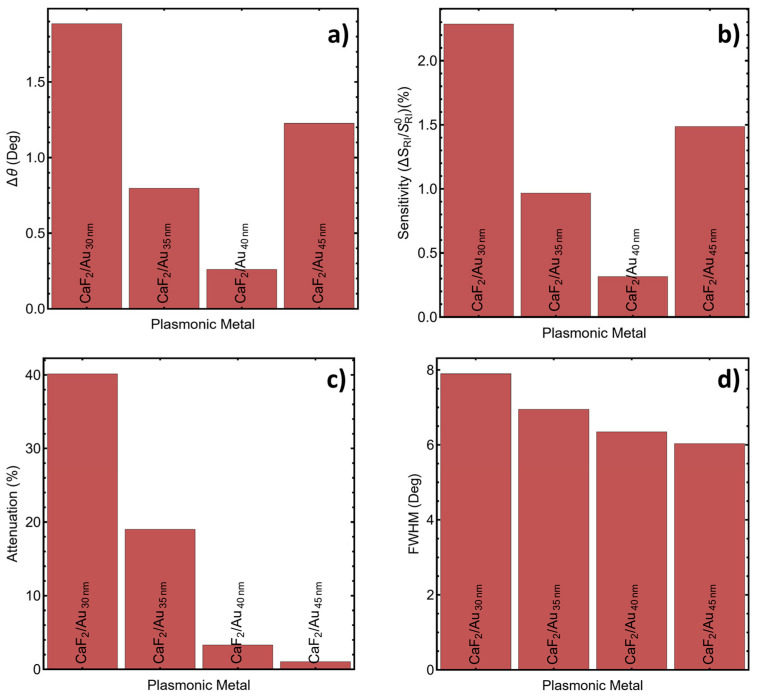
Effect of Au thickness (30–45 nm) on CaF_2_-coupled SPR performance under the modeled 1 ng/mL sensing condition: (**a**) resonance-angle shift Δθ, (**b**) sensitivity enhancement, (**c**) resonance attenuation, and (**d**) FWHM (Tables S5 and S6).

The same trend is reflected in the sensitivity enhancement values in Table S6 and Figure 4b. The 30 nm Au film yields the highest sensitivity enhancement (2.28%), while 35 nm decreases to 0.96% and 40 nm to 0.31%. Although the 45 nm case increases to 1.48% relative to 40 nm, its absolute angular shift remains extremely small (Δθ = 0.01°), which limits practical detectability in angle-interrogation measurements. These results support the physical expectation that sensitivity is maximized when the Au-layer thickness provides efficient plasmon excitation while still allowing substantial field penetration into the sensing region.

The remaining metrics clarify the trade-off between resonance strength and angular resolution. Figure 4c and Table S6 show that resonance attenuation decreases sharply with thickness, from 40.15% at 30 nm to 1.03% at 45 nm. This indicates that the dip becomes progressively shallower as the Au layer thickens, consistent with reduced coupling efficiency and weaker energy transfer into the plasmon mode. At the same time, Figure 4d shows that the resonance linewidth narrows with increasing thickness: FWHM decreases from 7.89° (30 nm) to 6.03° (45 nm). A narrower dip can improve the precision of resonance tracking, but, in this case, the gain in linewidth is accompanied by a strong loss in resonance depth and, more critically, by a collapse of Δθ. Therefore, the thickness that minimizes FWHM is not the most appropriate choice for ultralow-RI sensing because it does not preserve adequate angular sensitivity.

Considering the combined requirements of measurable resonance depth, a resolvable angular shift, and acceptable linewidth, the 30 nm Au film provides the most favorable balance among the tested cases for the CaF_2_-based configuration. It delivers the largest Δθ and the highest sensitivity enhancement while maintaining a pronounced SPR dip that can be robustly localized. This result identifies 30 nm as the working plasmonic thickness for the subsequent multilayer optimization, where the goal is no longer to tune the metal film alone but to evaluate how additional dielectric and 2D overlayers modify the response of the adopted CaF_2_/Au platform.

### 3.3. Optimization of the Silicon Nitride Layer for Enhanced Plasmonic Sensing

**Figure 5:**
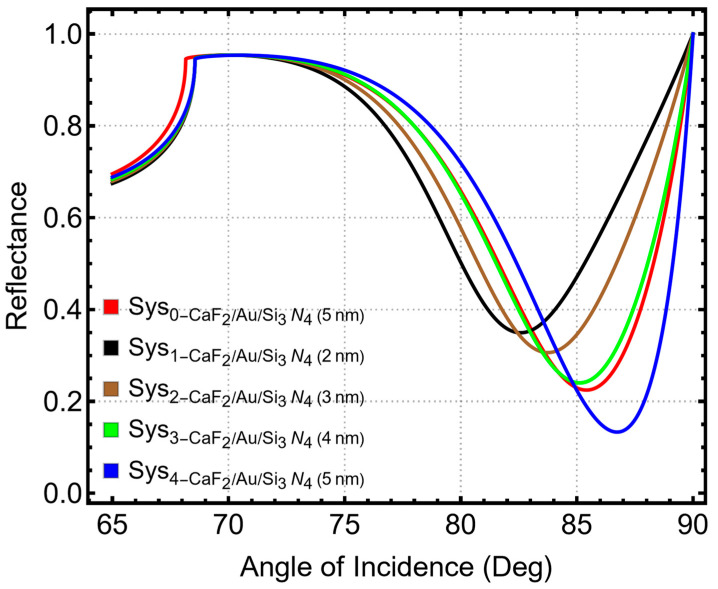
Angular reflectance spectra of the CaF_2_/Au/Si_3_N_4_ plasmonic stack computed for different silicon nitride overlayer thicknesses (2–5 nm), highlighting the modulation of the SPR dip depth and its angular position as the dielectric coating is tuned.

Figure 5 presents the angular reflectance spectra of the CaF_2_/Au/Si_3_N_4_ architecture for a Si_3_N_4_ thickness sweep in the nanometric range. All the curves show the characteristic SPR signature as a pronounced reflectance minimum at high incidence angles, which arises from phase matching between the incident optical mode and the surface plasmon polariton supported at the Au/dielectric interface. Increasing the Si_3_N_4_ thickness shifts the resonance minimum toward higher angles, consistent with dielectric loading of the plasmonic mode: the higher-index overlayer increases the effective refractive index in the near-metal region, thereby increasing the in-plane wavevector required for plasmon excitation and moving the resonance condition to larger incidence angles in the Kretschmann configuration. In addition to the angular displacement, the depth of the resonance dip varies across the sweep, indicating that the Si_3_N_4_ coating also alters coupling efficiency and the partitioning of energy between radiative leakage and absorptive loss in the metal. These thickness-dependent changes motivate an optimization based on quantitative readout metrics rather than relying only on visual inspection of the resonance dip.

The simulated multilayer stacks and sensing conditions associated with Sys0–Sys4 are summarized in Table S8. Sys_0_ corresponds to the CaF_2_/Au/Si_3_N_4_ configuration in water, whereas Sys_1_–Sys_4_ correspond to the CaF_2_/Au/Si_3_N_4_ platform under the modeled 1 ng/mL sensing condition with Si_3_N_4_ overlayer thicknesses of 2, 3, 4, and 5 nm, respectively. This mapping defines the thickness-varied structures used to extract the resonance position and performance metrics discussed below.

The thickness dependence of the resonance position is compactly captured in Figure S4, which summarizes the SPR peak angle as a function of Si_3_N_4_ thickness. The resonance angle increases monotonically from 82.57° at 2 nm to 86.73° at 5 nm, with intermediate values of 83.74° (3 nm) and 85.11° (4 nm), in agreement with the peak positions reported in Table S9. Table S10 further contextualizes this trend by reporting the same SPR peak angles for Sys_1_–Sys_4_, alongside the real part of the Si_3_N_4_ refractive index used in the simulations, clarifying the optical basis of the tuning: the observed angular shift follows directly from the dielectric contribution of the Si_3_N_4_ overlayer to the plasmon dispersion and the corresponding phase-matching condition. Together, Figure 5 and Figure S4 establish that Si_3_N_4_ thickness provides a direct handle to set the operating angle, while Tables S9 and S10 connect that tuning to the assumed optical constants and the extracted resonance positions.

**Figure 6:**
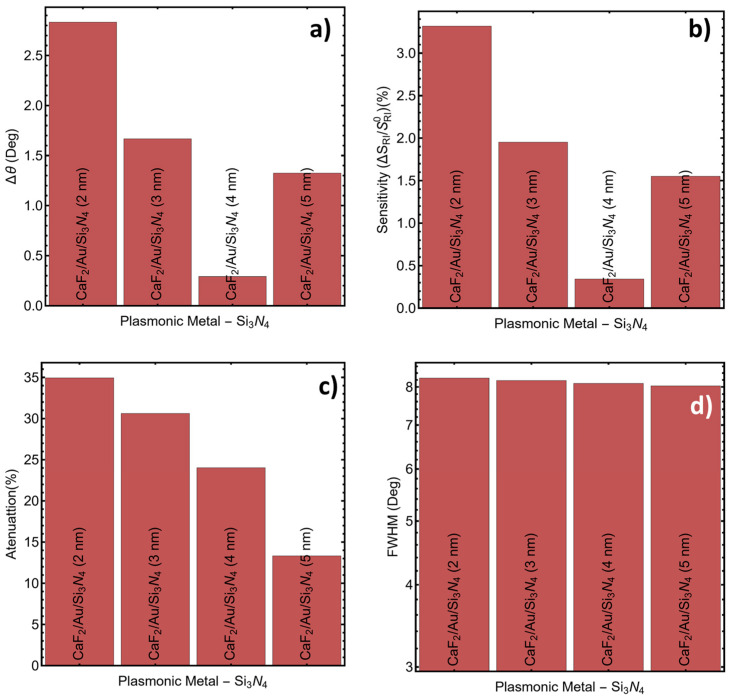
Key angular SPR performance metrics for the CaF_2_/Au/Si_3_N_4_ stacks with different Si_3_N_4_ thicknesses (Sys1–Sys4): (**a**) resonance-angle shift Δθ, (**b**) sensitivity enhancement, (**c**) resonance attenuation, and (**d**) FWHM.

Performance comparisons across thicknesses are quantified in Figure 6a–d and Table S9 using resonance angular shift (Δθ), sensitivity enhancement, attenuation, and FWHM. Figure 6a shows that Δθ is largest for the 2 nm coating and decreases markedly at 4 nm before partially recovering at 5 nm. Table S9 reports Δθ values of 2.83° (Sys_1_, 2 nm), 1.66° (Sys_2_, 3 nm), 0.29° (Sys_3_, 4 nm), and 1.32° (Sys_4_, 5 nm). The non-monotonic dependence indicates that the overlayer modifies not only the absolute resonance position but also the field distribution and coupling condition in a way that changes how strongly the resonance responds to the analyte perturbation at the outer interface. The same trend is reflected in Figure 6b, where the sensitivity enhancement is highest at 2 nm and lowest at 4 nm, with values of 3.31% (Sys_1_), 1.95% (Sys_2_), 0.34% (Sys_3_), and 1.55% (Sys_4_) in Table S9. Within the explored range, the 2 nm Si_3_N_4_ thickness yields the strongest angular response under the effective-refractive-index condition associated with 1 ng/mL, whereas 4 nm produces a weak shift and limited sensitivity enhancement.

Figure 6c provides a complementary perspective by tracking attenuation at resonance, which decreases with increasing Si_3_N_4_ thickness from 34.94% (2 nm) to 30.62% (3 nm), 24.02% (4 nm), and 13.31% (5 nm), as listed in Table S9. This reduction indicates that thicker Si_3_N_4_ coatings weaken the dissipative resonance signature in this configuration, consistent with altered impedance matching and a redistribution of electromagnetic energy between the metal and dielectric regions. Figure 6d shows that the FWHM varies only slightly across the thicknesses, remaining close to 8°, with Table S9 reporting 8.25° (2 nm), 8.18° (3 nm), 8.09° (4 nm), and 8.02° (5 nm). The modest narrowing with increasing thickness suggests only a limited change in resonance sharpness compared with the pronounced differences observed in Δθ and sensitivity enhancement.

Overall, the combined evidence from Figure 5 and Figure 6a–d and Figure S4 and Tables S8–S10 indicates a clear trade-off between angular response and resonance attenuation within the CaF_2_/Au/Si_3_N_4_ screening stage. Under the modeled 1 ng/mL condition and within the 2–5 nm design window, the 2 nm Si_3_N_4_ overlayer provides the largest Δθ and the highest sensitivity enhancement while maintaining an FWHM comparable to the thicker coatings. Increasing Si_3_N_4_ thickness shifts the operating angle upward and reduces attenuation, with only minor changes in FWHM, but does not maximize the angular response in this partial-stack configuration. This outcome therefore defines the Si_3_N_4_-only trend prior to incorporation of the outer nanomaterial overlayer used in the final multilayer architecture.

### 3.4. Optimization of the Nanomaterial for Enhanced Plasmonic Sensing

**Figure 7:**
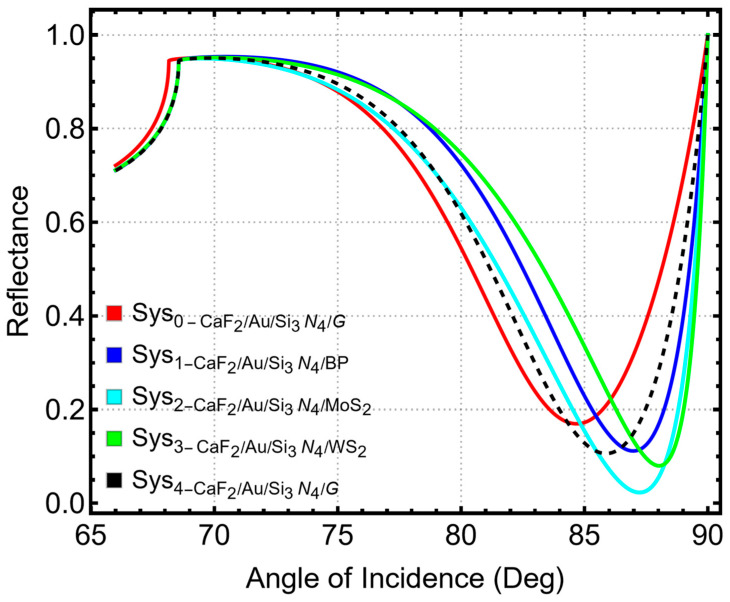
Angular reflectance spectra of the CaF_2_/Au/Si_3_N_4_ plasmonic stack functionalized with different nanomaterials (BP, MoS_2_, WS_2_, and graphene), showing the effect of the nanomaterial layer on the SPR dip position and depth.

Figure 7 compares the angular reflectance spectra of the CaF_2_/Au/Si_3_N_4_ platform functionalized with different nanomaterial layers under the modeled 1 ng/mL sensing condition. All the cases exhibit a well-defined SPR minimum, confirming that plasmon excitation is preserved after introducing the nanomaterial coating. However, the resonance position and dip depth differ among materials, indicating that the added layer modifies the effective optical environment and the coupling condition at the metal/dielectric boundary. Relative to graphene and black phosphorus, transition-metal dichalcogenides (MoS_2_ and WS_2_) shift the resonance toward higher angles and yield a more pronounced minimum, consistent with stronger dielectric loading and enhanced field–matter interaction in the near-surface region. The spectral separation among curves supports the use of nanomaterial selection as an additional degree of freedom for tuning the operating angle and the strength of the resonance response beyond the Si_3_N_4_ optimization.

Table S11 defines the systems and modeled sensing conditions used for the nanomaterial comparison. Sys_0_ corresponds to the CaF_2_/Au/Si_3_N_4_/graphene configuration in water, whereas Sys_1_–Sys_4_ correspond to the effective-refractive-index condition associated with 1 ng/mL for BP, MoS_2_, WS_2_, and graphene, respectively. This mapping establishes the set of nanomaterial-functionalized structures used to extract resonance positions and performance metrics from the spectra in Figure 7 and to quantify the comparative sensing response discussed below.

**Figure 8:**
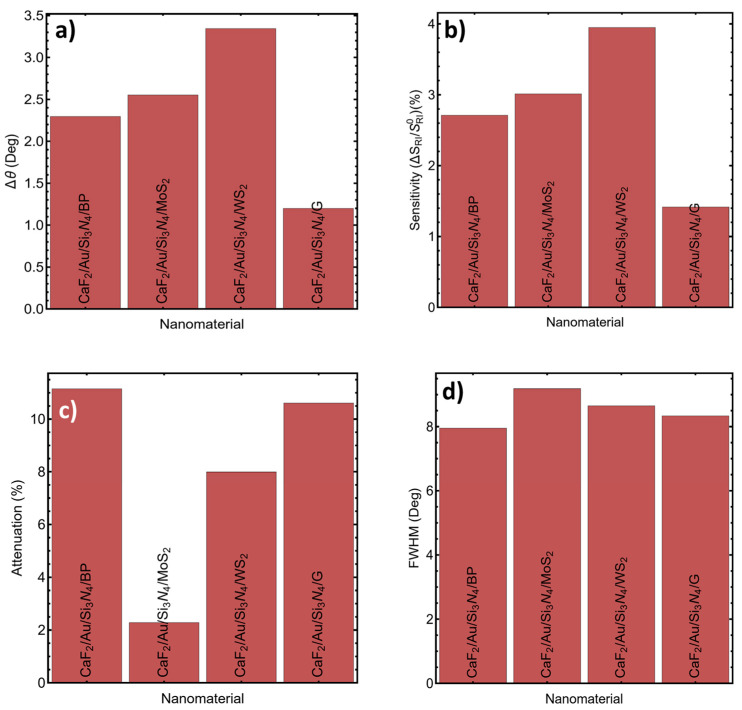
Key angular SPR performance metrics for different nanomaterial-functionalized CaF_2_/Au/Si_3_N_4_ stacks under the modeled 1 ng/mL sensing condition: (**a**) resonance angular shift Δθ, (**b**) sensitivity enhancement, (**c**) attenuation at resonance, and (**d**) FWHM.

The quantitative impact of nanomaterial choice is summarized by the metric panels in Figure 8a–d and the values reported in Table S12. Figure 8a shows that the angular shift Δθ is maximized for WS_2_, followed by MoS_2_ and BP, while graphene yields the smallest shift. Table S12 reports Δθ = 2.29° for BP (Sys_1_), 2.55° for MoS_2_ (Sys_2_), 3.34° for WS_2_ (Sys_3_), and 1.20° for graphene (Sys_4_). The same ordering appears in Figure 8b, where sensitivity enhancement reaches its maximum for WS_2_ at 3.94%, followed by MoS_2_ at 3.01% and BP at 2.71%, whereas graphene provides 1.41% (Table S12). These results indicate that, within this platform and modeled 1 ng/mL condition, WS_2_ provides the strongest amplification of the angular response, consistent with enhanced electromagnetic-field confinement and/or stronger perturbation of the plasmon dispersion at the sensing interface.

Figure 8c places these gains in the context of attenuation, which reflects the resonance depth and thus the strength of energy transfer into the plasmonic mode. MoS_2_ exhibits the lowest attenuation (2.28%), whereas BP and graphene show higher attenuation at 11.15% and 10.61%, respectively, with WS_2_ intermediate at 7.98% (Table S12). This indicates that maximizing angular sensitivity does not necessarily coincide with maximizing resonance attenuation; WS_2_ provides the highest Δθ and sensitivity enhancement despite not producing the deepest resonance signature among the compared coatings. Figure 8d shows that the FWHM remains in a relatively narrow range across the materials, from 7.94° (BP) to 9.18° (MoS_2_), with WS_2_ at 8.65° and graphene at 8.33% (Table S12). The moderate differences in FWHM suggest that the primary performance discrimination among the nanomaterials in this dataset arises from Δθ and sensitivity enhancement rather than substantial changes in resonance linewidth.

The dependence of resonance position on the nanomaterial optical constants is further clarified by Figure S5 and Table S13. Figure S5 relates the SPR peak position to the real part of the nanomaterial refractive index, showing that higher Re(n) generally pushes the resonance toward higher angles, with WS_2_ exhibiting the largest peak position. Table S13 reports Re(n) values of 3.50 (BP), 5.08 (MoS_2_), 4.90 (WS_2_), and 3.00 (graphene), with corresponding SPR peak positions of 86.97°, 87.23°, 88.02°, and 85.88°, respectively. The ordering of peak angles is therefore consistent with dielectric loading by the nanomaterial layer, while deviations from a strictly linear dependence can be rationalized by the presence of non-negligible loss, reflected in the reported imaginary parts in Table S13, and by the fact that coupling depends on the full multilayer dispersion rather than on Re(n) alone. Together, Figure 7 and Figure 8a–d and Figure S5 and Tables S11–S13 show that WS_2_ provides the most favorable optical trade-off in this specific nanomaterial screening step, delivering the largest angular shift and sensitivity enhancement with moderate linewidth and attenuation under the modeled 1 ng/mL condition. This comparison therefore identifies WS_2_ as the strongest optical performer within the tested set, but it does not by itself define the final practical material choice. Within the broader theoretical framework of this study, BP was retained as an ultrathin overlayer of interface-engineering interest because its optical and dielectric characteristics remain relevant to multilayer SPR design, while its practical use must still be considered together with its known stability limitations.

### 3.5. Sensor Performance

**Figure 9:**
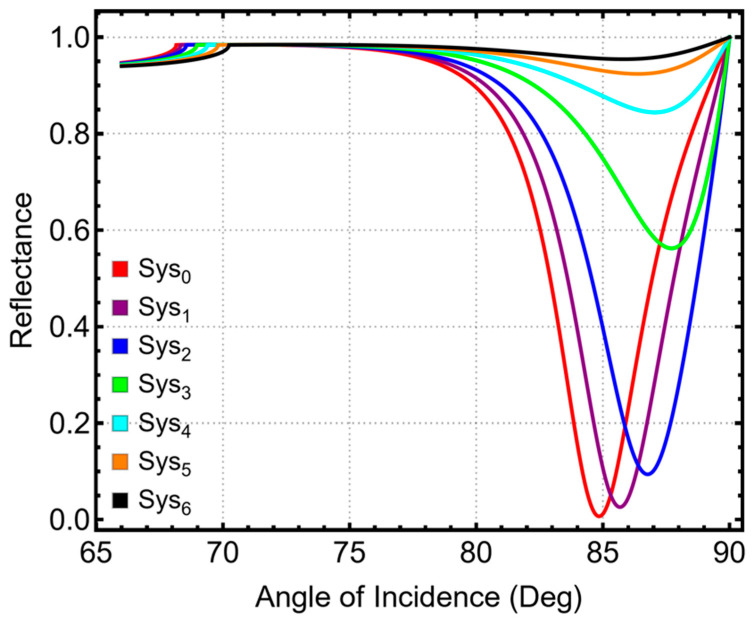
Angular reflectance spectra for Sys_0_–Sys_6_, showing the evolution of the SPR minimum under increasing modeled refractive-index conditions. Sys_0_: water; Sys_1_: DIW; Sys_2_–Sys_6_: effective-refractive-index cases associated with 1–5 ng/mL.

The concentration-dependent analysis in this section is performed for the final CaF_2_/Au/Si_3_N_4_/BP multilayer configuration adopted in the manuscript. In this full-stack architecture, the Si_3_N_4_ thickness corresponds to the value used together with the BP overlayer in the final sensor design rather than to the Si_3_N_4_-only screening outcome discussed in Section 3.3. The reflectance–angle spectra in Figure 9 show a clear SPR signature, with a pronounced dip whose angular position depends on the refractive-index condition assigned to the sensing medium. Across the modeled low-concentration cases, the effective-refractive-index values increase monotonically (Table S14: 1.3337 at 1 ng/mL to 1.3485 at 5 ng/mL), which, in the present optical framework, increases the dielectric loading seen by the multilayer structure and shifts the resonance condition to new angles. In the low-to-moderate cases, the resonance minimum remains in the high-angle region (around the mid-80° range), consistent with a prism-coupled SPR configuration operating close to total internal reflection. At the highest modeled case (Sys_6_), the resonance shifts to a much lower angle, and the dip becomes substantially broader, indicating a strong perturbation of the optical response and higher damping, which is also reflected in the large FWHM reported in Table S16. This pronounced broadening also indicates reduced robustness in resonance tracking at the highest assigned refractive-index condition and places Sys_6_ near the edge of the most stable operating regime of the structure.

**Figure 10:**
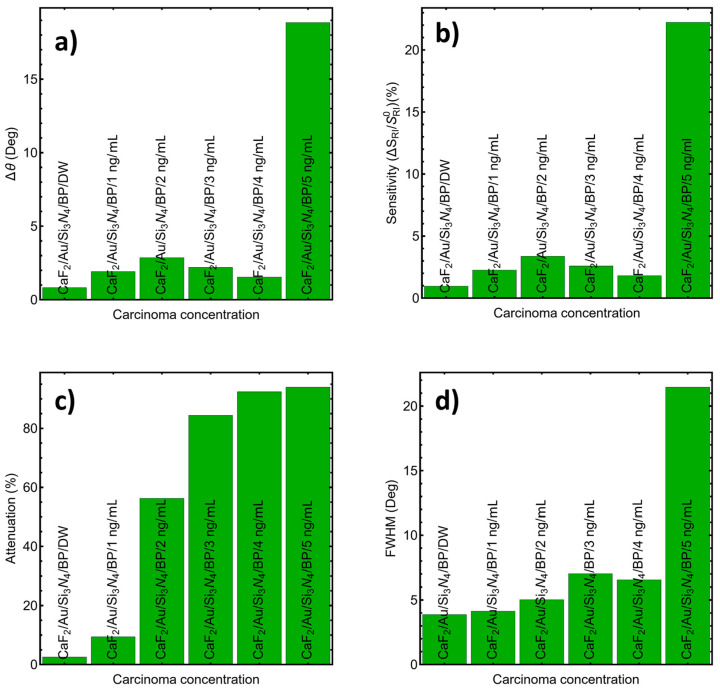
Performance of the SPR nanosensor across the modeled 1–5 ng/mL conditions, (**a**) resonance-angle shift Δθ, (**b**) sensitivity, (**c**) attenuation, and (**d**) FWHM, highlighting the sensitivity–linewidth trade-off at higher refractive-index loading.

Figure 10 consolidates the concentration-dependent response into four complementary metrics that together describe both the magnitude of the SPR shift and the quality of the resonance used for angle interrogation. In Figure 10a, the angular shift Δθ increases across the modeled concentration conditions, reflecting the monotonic increase in the effective-refractive-index values listed in Table S14 and confirming that the resonance condition is progressively perturbed under higher optical loading. This increase in Δθ directly drives the trend in Figure 10b, where sensitivity rises because the sensor converts larger refractive-index increments into larger angular displacements. At the same time, Figure 10c shows that attenuation increases markedly at the higher modeled conditions, indicating stronger coupling losses and/or increased damping in the plasmonic mode as the resonance becomes deeper and more dissipative. Consistent with this damping, Figure 10d shows a pronounced broadening of the resonance, with the FWHM increasing toward the highest case, which implies reduced sharpness of the resonance minimum and therefore a potential penalty in angular readout precision despite the larger shift. Under these highest loading conditions, the increase in angular displacement must therefore be interpreted together with the marked linewidth broadening since a larger shift alone does not imply a more reliable sensing state.

Taken together, Figure 10a,b show larger angular displacements under the higher modeled conditions, while Figure 10c,d indicate that this response is accompanied by higher loss and broader linewidth. The concentration-dependent analysis therefore identifies the lower portion of the modeled range as the most reliable operating window of the proposed structure, whereas the highest assigned refractive-index condition should be interpreted more cautiously because resonance broadening increasingly compromises angular readout robustness.

### 3.6. Sensor Metrics

**Figure 11:**
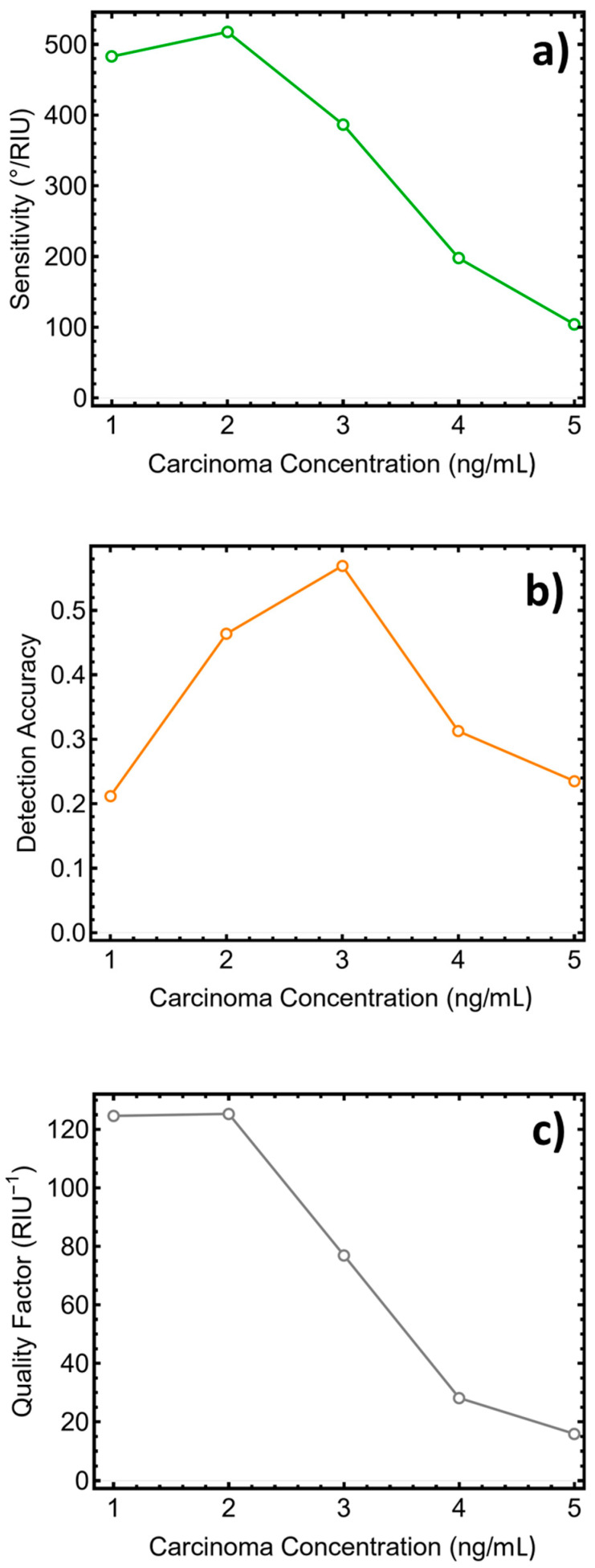
Secondary metrics of the SPR biosensor derived from the modeled effective-RI conditions, (**a**) refractive-index sensitivity, (**b**) detection accuracy, and (**c**) quality factor, evaluated across the 1–5 ng/mL modeled range (see Tables S17 and S18).

**Figure 12:**
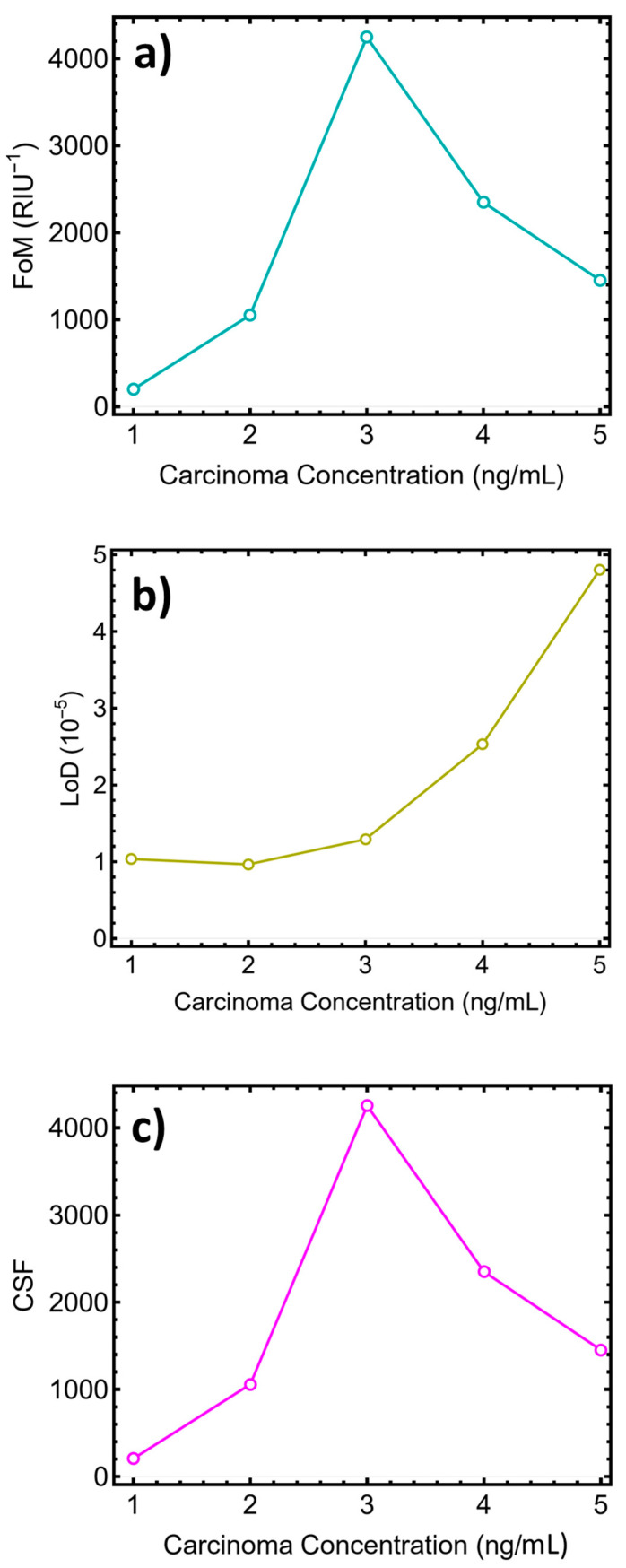
Composite performance indicators derived from the modeled effective-RI conditions across the 1–5 ng/mL range, (**a**) FoM, (**b**) LoD, and (**c**) CSF, highlighting the operating window that maximizes discrimination while maintaining a comparatively narrow resonance (see Tables S17 and S19).

The analysis examines how the assigned effective refractive index (RI) conditions are reflected in secondary metrics that capture both the magnitude of the SPR response and the reliability of angular readout. In this context, sensitivity quantifies the angular response to refractive-index variation, whereas detection accuracy (DA) and quality factor (QF) primarily reflect the sharpness of the resonance minimum and the expected precision of angular localization. Figure of merit (FoM) and contrast signal factor (CSF) are composite indicators that combine responsivity with resonance quality, while the reported LoD should be interpreted as a theoretical comparative indicator within the present numerical framework. The RI values assigned to each concentration step and the corresponding SPR peak positions are summarized in Table S17, which establishes the concentration–RI–resonance mapping used to compute the performance indicators reported in Tables S18 and S19 and plotted in Figure 11 and Figure 12.

Figure 11a shows that refractive-index sensitivity varies nonlinearly across the modeled 1–5 range, with the highest sensitivity occurring at low concentrations and a progressive decline toward the higher assigned RI conditions. This behavior indicates that the structure does not operate in a uniform linear sensing regime across the full modeled range, and that the most reliable calibration window is concentrated in the lower portion of the assigned RI conditions. The same regime dependence appears in detection accuracy (Figure 11b), which increases from the lowest concentration to an intermediate maximum and then decreases at higher concentrations, indicating that resonance broadening and loss increasingly limit the precision of locating the SPR minimum even when RI shifts remain present. This interpretation is reinforced by the quality factor trend in Figure 11c, which drops substantially at higher modeled conditions, reflecting degradation in resonance sharpness that reduces the practical resolution of angle interrogation. The underlying numerical values for sensitivity, detection accuracy, and quality factor are reported in Table S18, and their trends should be interpreted together with Table S17 because the derived metrics depend directly on how the SPR peak position evolves under the assigned RI conditions.

Figure 12 evaluates composite indicators that combine responsivity and resonance sharpness. The figure of merit in Figure 12a rises sharply to a maximum at the intermediate concentration and then decreases, reflecting the balance between angular response and resonance broadening implied by Figure 11a–c. The contrast signal factor in Figure 12c follows a similar peaked dependence, indicating that the strongest discrimination between concentration states occurs when the resonance remains sufficiently deep and narrow while still providing substantial angular separation. In contrast, the limit of detection in Figure 12b worsens toward higher concentrations, consistent with increased damping and linewidth inflation reducing the effective measurement resolution. Because instrumental angular resolution and noise floor were not explicitly modeled here, the reported LoD should be interpreted cautiously as a comparative theoretical indicator rather than as a directly transferable experimental limit. The quantitative values supporting these trends are summarized in Table S19, and their physical meaning is anchored by the RI–SPR-peak correlation reported in Table S17.

Taken together, Figure 11 and Figure 12 and Tables S17–S19 indicate that discrimination performance is most favorable in the low-to-intermediate portion of the modeled range, where FoM and CSF remain high while resonance sharpness is still preserved. By contrast, at the upper end of the modeled range, the drop in quality factor and the rise in LoD indicate that broadening and loss dominate the optical readout. Accordingly, the full 1–5 ng/mL interval should not be interpreted as a uniformly linear calibration range but rather as a modeled operating span with metric-dependent trade-offs and a more favorable low-to-intermediate response window.

### 3.7. Spatial Distribution of the Electric Field and Sensing Interface Performance

**Figure 13:**
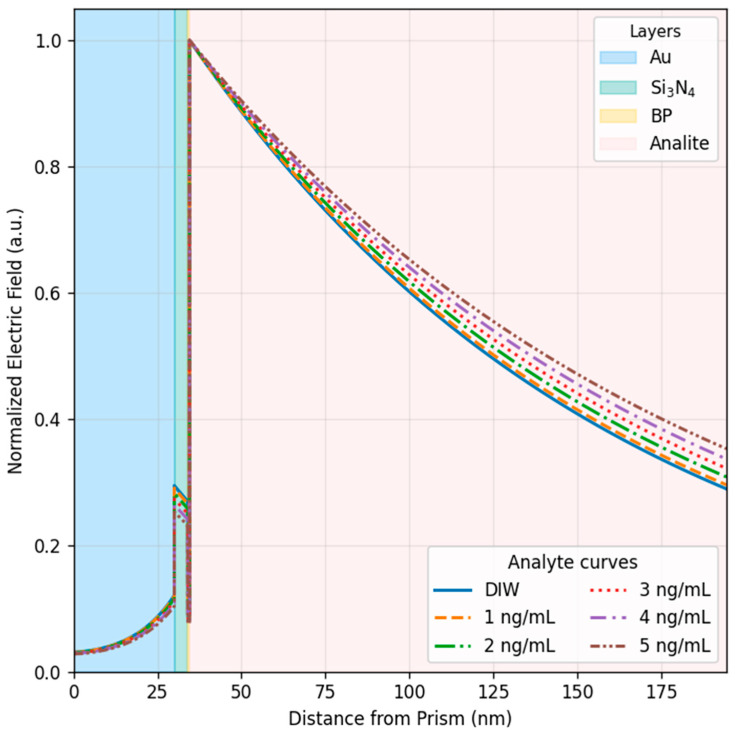
Normalized electric-field distribution across the Au/Si_3_N_4_/BP multilayer for DIW and the modeled effective-refractive-index conditions associated with 1–5 ng/mL (Tables S14 and S17).

Figure 13 describes how the electromagnetic field associated with the excited plasmonic mode is distributed across the multilayer stack and into the sensing medium, which is the physical basis for refractive-index interrogation. The shaded regions mark the successive layers (Au, Si_3_N_4_, BP, and sensing medium), and the plotted curves show the normalized electric-field amplitude as a function of distance measured from the prism side. A rapid variation is observed across the metal region, followed by a strong field discontinuity at the metal/dielectric boundary and a pronounced evanescent tail that penetrates into the sensing medium. This spatial profile is consistent with prism-coupled SPR excitation, where energy is concentrated near the metal interface and decays exponentially into the outer medium; therefore, the effective sensing volume is governed primarily by the near-surface region adjacent to the BP overlayer.

The set of curves corresponding to DIW and the modeled 1–5 ng/mL conditions shows that increasing assigned refractive-index loading produces a systematic increase in the field amplitude within the outer sensing region at a given distance from the interface. This trend is consistent with the effective-refractive-index values used in the modeling (Tables S14 and S17) because higher assigned RI modifies the boundary conditions and the mode confinement, increasing the fraction of optical energy stored in the superstrate and altering the coupling balance across the stack. In practical terms, the stronger field maintained within the sensing region implies enhanced interaction between the plasmonic mode and the surrounding medium, which supports the larger resonance perturbations and derived metrics reported earlier (Figure 11 and Figure 12; Tables S18 and S19). The figure also indicates that the presence of the Si_3_N_4_ spacer and BP overlayer does not eliminate the evanescent penetration but instead shapes the field localization so that substantial intensity remains available at the sensing interface, which is essential for detecting small effective-RI changes within the present numerical framework.

### 3.8. Performance Benchmarking Against Reported SPR Biosensor Architectures

Table 1 positions the proposed CaF_2_/Au/Si_3_N_4_/black phosphorus (BP) configuration against representative SPR biosensors reported in the recent literature [33,40,41,42,43], using sensitivity, quality factor (QF), and detection accuracy (DA) as the main comparison axes. Because the benchmarked reports include both angular and spectral interrogation schemes, sensitivity is listed in the original units reported by each study (e.g., °/RIU or THz/RIU), and direct cross-unit equivalence is not implied. The benchmark set spans different material strategies, including graphene-, ZnSe-, Franckeite-, and TiO_2_-assisted multilayers [33,40,41,42,43], thereby providing a broader context for positioning the present CaF_2_/Au/Si_3_N_4_/BP design.

Within this comparison, the most relevant outcome is that the proposed architecture is competitively positioned in the low-concentration operating regime. A representative reported multilayer sensor at 3 ng/mL achieves 481.29°/RIU with QF = 108.27 RIU^−1^ and DA = 0.80 [33,40,41,42,43], whereas the present structure reaches 482.82°/RIU at 1 ng/mL and 517.62°/RIU at 2 ng/mL, together with QF values above 120 RIU^−1^ at both points. This comparison is important because high sensitivity alone is not sufficient for robust angular interrogation; the simultaneous preservation of high QF indicates that the resonance remains comparatively sharp and therefore more suitable for reliable peak localization.

A further strength of the present work is that performance is reported at multiple modeled concentration conditions rather than at a single operating point. This makes it possible to identify the most favorable optical window of the structure and to observe how the metrics evolve as effective-refractive-index loading increases. The results show that the CaF_2_/Au/Si_3_N_4_/BP platform performs most favorably in the low-concentration portion of the modeled range, where both sensitivity and QF remain high and DA increases from 0.211 at 1 ng/mL to 0.463–0.569 at 2–3 ng/mL. At higher assigned RI conditions, sensitivity drops markedly, and the accompanying loss of resonance quality becomes more evident. The benchmarking therefore supports the view that the principal strength of the proposed architecture lies not in uniform performance across the entire range but in a favorable low-level operating window.

Beyond the tabulated metrics, the proposed platform also reflects a coherent multilayer design rationale. The CaF_2_ prism provides practical coupling conditions, the Au layer sustains plasmon excitation, the Si_3_N_4_ spacer helps to moderate damping while preserving field confinement, and the BP overlayer modifies the optical response at the sensing interface. In this sense, the contribution of the present work is not only the competitive values reported in Table 1 but also the stepwise optimization pathway that identifies how these elements jointly shape the final response. Overall, the benchmarking supports the relevance of the proposed architecture as a preliminary refractive-index-sensing platform whose strongest performance is concentrated in a favorable low-level operating window. Its practical translation into biosensing applications would require subsequent experimental validation, including surface biofunctionalization and BP stabilization under liquid-phase conditions.

## 4. Limitations

This work is a theoretical analysis based on the TMM under idealized assumptions of planar laterally uniform layers with nominal optical constants. Accordingly, the reported metrics should be interpreted as upper-bound optical design targets rather than guaranteed experimental outcomes since fabrication tolerances, interfacial roughness, and defects in ultrathin coatings can broaden the SPR dip and reduce resonance quality.

Practical implementation of the proposed multilayer platform would also require attention to material stability and biointerface design. Black phosphorus (BP) is represented here through literature-based optical constants as an idealized ultrathin overlayer, without explicitly distinguishing among pristine, oxidized, encapsulated, or otherwise protected states. In real liquid-phase sensing, BP stabilization, possible adhesion layers, and additional biochemical interface layers would modify the outer optical stack and could shift the resonance condition relative to the present configuration.

From a biosensor standpoint, realistic operation would further depend on receptor immobilization chemistry, nonspecific-binding suppression, controlled liquid handling, and repeatable performance in complex biofluids. Integration with a prism-coupled flow cell or microfluidic arrangement would provide controlled sample delivery, reduced sample volume, and improved repeatability during liquid-phase measurements. Such an implementation would also facilitate washing, regeneration, and sequential exposure steps, although it would need to be combined with surface biofunctionalization and BP stabilization to remain compatible with realistic bioassay operation. These effects were not explicitly included in the present model. In addition, the concentration-dependent cases are treated as effective-refractive-index conditions rather than as a target-specific interfacial binding model. At higher optical loading, the SPR dip becomes broader and shallower, which can reduce the robustness of resonance tracking and contribute to non-monotonic angular behavior. The reported LoD should also be interpreted cautiously since instrumental angular resolution and noise floor were not explicitly incorporated. Future work should therefore focus on experimental validation, fabrication variability, BP stabilization, and operation under realistic liquid-phase-assay conditions.

## 5. Conclusions

This study used transfer-matrix simulations to evaluate a prism-coupled multilayer SPR sensor and showed that its response depends on the coupled choice of prism, Au thickness, dielectric spacer, and outer nanomaterial overlayer. The stepwise optimization identified CaF_2_ as the practical working prism, 30 nm as the preferred Au thickness, and a favorable low-concentration operating window for the final CaF_2_/Au/Si_3_N_4_/BP configuration.

Under the adopted effective-refractive-index conditions, the structure reached 482.82°/RIU at 1 ng/mL and 517.62°/RIU at 2 ng/mL, together with QF values above 120 RIU^−1^, indicating high angular responsivity with comparatively sharp resonance features in the low-concentration regime. At higher assigned RI conditions, resonance broadening reduced readout robustness, so the full 1–5 ng/mL range should not be interpreted as a uniformly linear calibration interval.

Overall, the results support the proposed architecture as a preliminary refractive-index-sensing platform with competitive low-level performance. Practical translation would require experimental validation, BP stabilization, surface biofunctionalization, nonspecific-binding control, and realistic liquid-phase implementation.

## Figures and Tables

**Table 1:** Comparison of the proposed CaF_2_/Au/Si_3_N_4_/BP SPR biosensor with representative reported SPR designs in terms of structure, analyte concentration, angular sensitivity, quality factor, and detection accuracy.

Ref.	Structure	Conc. (ng/mL)	Sensitivity (°/RIU)	QF	DA
Rafighirami et al. [33]	Au/SiO_2_/Graphene	3	4.306 THz/RIU	53.9	--
Juwel et al. [40]	CaF_2_/MgO/Ag/ZnSe/Graphene	5	393.83	90.11	0.22
Kumar et al. [41]	CaF_2_/Cu/BP/Franckeite	5	348.07	--	--
Khodiae et al. [42]	BK7/Ag/Ni/ZnSe	--	163.63	--	0.10
Tene et al. [43]	CaF_2_/Cu/TiO_2_/Graphene	3	481.29	108.27	0.80
This work	CaF_2_/Au/Si_3_N_4_/Black Phosphorus	1	482.82	124.60	0.211
This work	CaF_2_/Au/Si_3_N_4_/Black Phosphorus	2	517.62	125.28	0.463
This work	CaF_2_/Au/Si_3_N_4_/Black Phosphorus	3	386.59	76.90	0.569
This work	CaF_2_/Au/Si_3_N_4_/Black Phosphorus	4	197.62	28.15	0.312
This work	CaF_2_/Au/Si_3_N_4_/Black Phosphorus	5	104.10	15.88	0.235

## Data Availability

Data will be made available on request.

## Supplementary Materials

The following supporting information can be downloaded at: https://www.mdpi.com/article/10.3390/bios16040198/s1.
