# Supplementary material for: Modeling an SPR Sensor for Carcinoma-Related Refractive-Index Detection: The Case of CaF2/Au/Si3N4/BP Multilayer System
Source: Biosensors (Basel). 2026 Apr 1;16(4):198. doi: 10.3390/bios16040198 (PMC13113593; doi:10.3390/bios16040198)
Supplement: Supplementary file 1 [file biosensors-16-00198-s001.zip › biosensors-4210236-supplementary.pdf]

SUPPLEMENTARY INFORMATION

Modeling an SPR Sensor for Carcinoma-Related Refractive-Index Detection: The Case of CaF_2/Au/Si_3N_4/BP Multilayer System

Talia Tene [1,*], Martha Ximena Dávalos Villegas [2] and Cristian Vacacela Gomez [3,4,*]

[1] Department of Chemistry, Universidad Técnica Particular de Loja, Loja 110160, Ecuador
[2] Facultad de Ciencias, Escuela Superior Politécnica de Chimborazo (ESPOCH), Panamericana Sur km. 1 1/2, Riobamba 060155, Ecuador
[3] Department of Physics, University of Calabria, Via P. Bucci, Cubo 33C, 87036 Rende, Italy
[4] Universidad Ecotec, Km. 13.5 Samborondón, Samborondón EC092302, Ecuador
* Correspondence: tbtene@utpl.edu.ec (T.T.); cristianisaac.vacacelagomez@fis.unical.it (C.V.G.)

1. Computational Metrics and Transfer-Matrix Formulation

Sensor performance was assessed using the angular-interrogation metrics defined in Equations (S1)–(S7). The refractive-index sensitivity was computed from the resonance-angle response to a refractive-index variation:

$$S_{RI} = \frac{\Delta\theta}{\Delta n} \qquad \text{(S1)}$$

where Δθ is the resonance-angle shift and Δn is the corresponding refractive-index change. To quantify the improvement relative to a baseline condition (before analyte adsorption), the sensitivity enhancement was evaluated as

$$\Delta S_{RI}^{after} = \frac{(S_{RI}^{after} - S_{RI}^{before})}{S_{RI}^{before}} \qquad \text{(S2)}$$

The detection accuracy was defined by the ratio between the angular shift and the full width at half maximum of the SPR dip:

$$DA = \frac{\Delta\theta}{FWHM} \qquad \text{(S3)}$$

and the quality factor was calculated as

$$QF = \frac{S_{RI}}{FWHM} \qquad \text{(S4)}$$

The figure of merit incorporated both the sensitivity and the resonance depth through the minimum reflectance at resonance:

$$FoM = \frac{S_{RI}(1 - R_{min})}{FWHM} \qquad \text{(S5)}$$

where R_{min} denotes the minimum normalized reflectance value of the SPR curve. The limit of detection was estimated by

$$LoD = \frac{\Delta n}{\Delta\theta} \times 0.005° \qquad \text{(S6)}$$

Finally, the combined sensitivity factor was obtained using the reflectance contrast between the off-resonant level and the resonance minimum:

$$CSF = \frac{S_{RI} \times (R_{max} - R_{min})}{FWHM} \qquad \text{(S7)}$$

where R_{max} is the reflectance level away from resonance.

The optical response required to extract θ, FWH, R_{min}, and R_{max} was computed using the transfer matrix method (TMM) [26,27]. The tangential electric and magnetic field components in the incident region were related to those in the last region of the multilayer stack through the global transfer matrix,

$$\begin{bmatrix} E_1 \\ H_1 \end{bmatrix} = M_{ij} \begin{bmatrix} E_{N-1} \\ H_{N-1} \end{bmatrix} \qquad \text{(S8)}$$

where M_{ij} is obtained from the ordered product of the layer characteristic matrices,

$$M_{ij} = \left[\prod_{k=2}^{N-1} M_k\right]_{ij} = \begin{bmatrix} M_{11} & M_{12} \\ M_{21} & M_{22} \end{bmatrix} \tag{S9}$$

Each layer matrix M_k is written as

$$M_k = \begin{bmatrix} \cos\beta_k & (-\mathrm{i}\sin\beta_k)/q_k \\ -\mathrm{i}\, q_k \sin\beta_k & \cos\beta_k \end{bmatrix} \tag{S10}$$

with the phase term and auxiliary parameter given by

$$\beta_k = \frac{2\pi d_k}{\lambda_0}\sqrt{\varepsilon_k - n_1^2 \sin^2\theta} \tag{S11}$$

$$q_k = \frac{\sqrt{\varepsilon_k - n_1^2 \sin^2\theta}}{\varepsilon_k} \tag{S12}$$

In Equations (10)–(12), λ_0 is the free-space wavelength, n1 is the refractive index of the first medium, ε_k is the dielectric constant of the k_{th} layer, θ is the incidence angle, and dk is the thickness of the k_{th} layer. A He–Ne wavelength of λ_0=633 nm was used to generate the angular reflectance spectra.

After determining the global matrix elements, the reflectance of the N-layer structure was calculated as

$$R = \left|\frac{(M_{11} + M_{12}\, q_N)q_1 - (M_{21} + M_{22}\, q_N)}{(M_{11} + M_{12}\, q_N)q_1 + (M_{21} + M_{22}\, q_N)}\right|^2 \tag{S13}$$

which yields the SPR curve R(θ) used for resonance-angle extraction and subsequent metric evaluation. All computations were performed for TM polarization using an angular sampling of 5×10^4 points.

2. Supplementary Figures

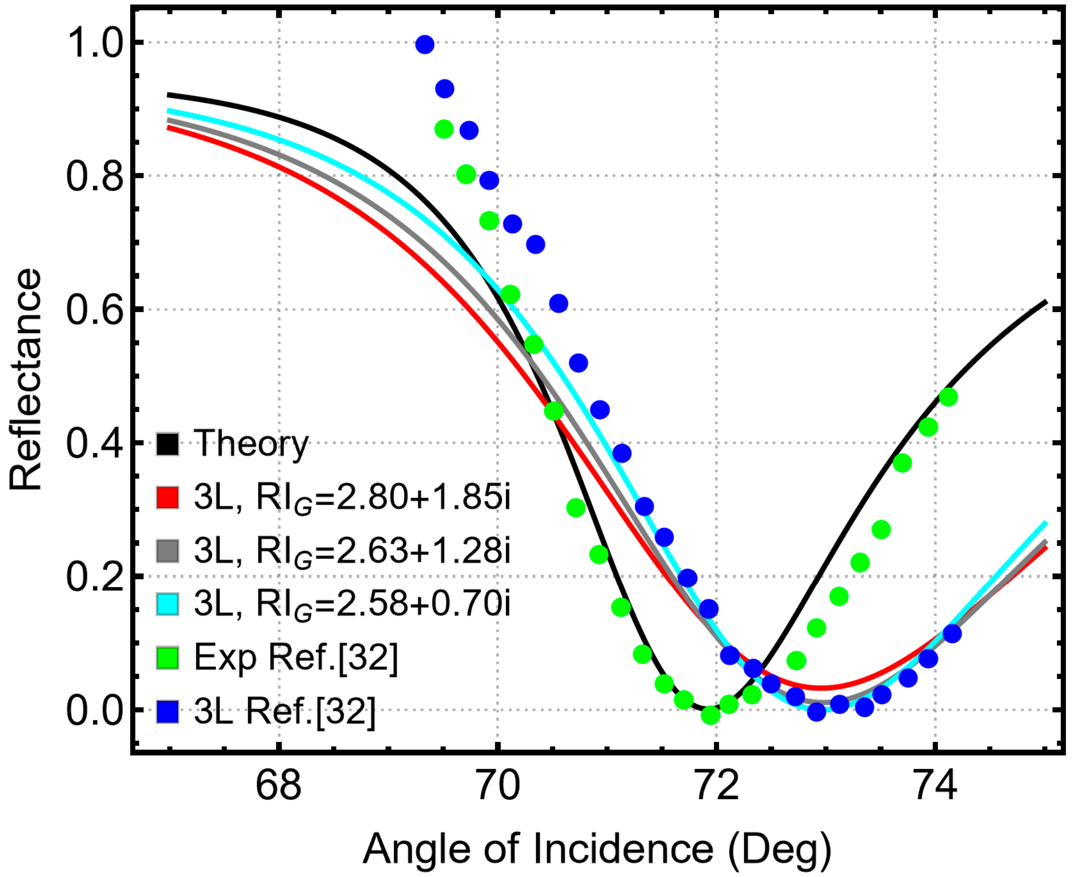

Figure S1. TMM validation: angle-dependent reflectance R(θ) of a three-layer model compared with experimental data from Ref. [32], illustrating the typical line shape and agreement with the literature.

3. Supplementary Tables

Table S1. Tested SPR systems for performance comparison. Each system varies in substrate and analyte configuration at 1 ng/mL carcinoma concentration.

Code	Full Name	Short Name
$Sys_{0\text{-}Bk7}$	BK_7/Gold/Water	BK_7/Au/H_2O
$Sys_{1\text{-}CaF2}$	CaF_2/Gold/Carcinoma-1 ng/mL	CaF_2/Au/Carcinoma-$_{1\ ng/mL}$
$Sys_{2\text{-}CsF}$	CsF/Gold/Carcinoma-1 ng/mL	CsF/Au/Carcinoma-$_{1\ ng/mL}$
$Sys_{3\text{-}SF6}$	SF_6/Gold/Carcinoma-1 ng/mL	SF_6/Au/Carcinoma-$_{1\ ng/mL}$
$Sys_{4\text{-}BK7}$	BK_7/Gold/Carcinoma-1 ng/mL	BK_7/Au/Carcinoma-$_{1\ ng/mL}$

Table S2. Optical properties and thicknesses of materials used in the SPR sensor simulations. Refractive indices are taken from references in the literature.

Material	Refractive Index	Thickness (nm)	Ref.
BK_7	1.5151	---	[34]
CaF_2	1.4329	---	[34]
CsF	1.4768	---	[34]
SF_6	1.7990	---	[34]
Gold (Au)	0.1378 + 3.6196i	45.0	[34]
Silicon Nitride (Si_3N_4)	2.0394	5.00	[35]
Black Phosphorus (BP)	3.5 + 0.01i	0.53	[36]
Water Medium (H_2O)	1.3300	---	[30-31-32]
Carcinoma (1 ng/mL)	1.3337	---	[33]

Table S3. Performance metrics of prism-based configurations: resonance position, angular shift, sensitivity, attenuation, and FWHM.

Code	SPR Peak Position	$\Delta\theta$ (Deg)	Sensitivity Enhancement (%)	Attenuation (%)	FWHM
$Sys_{1\text{-}CaF2}$	83.76	13.30	18.88	1.03	6.46
$Sys_{2\text{-}CsF}$	75.48	5.02	7.13	2.68	5.97
$Sys_{3\text{-}SF6}$	53.04	17.41	24.71	3.23	3.90
$Sys_{4\text{-}BK7}$	70.95	0.49	0.70	3.80	5.83

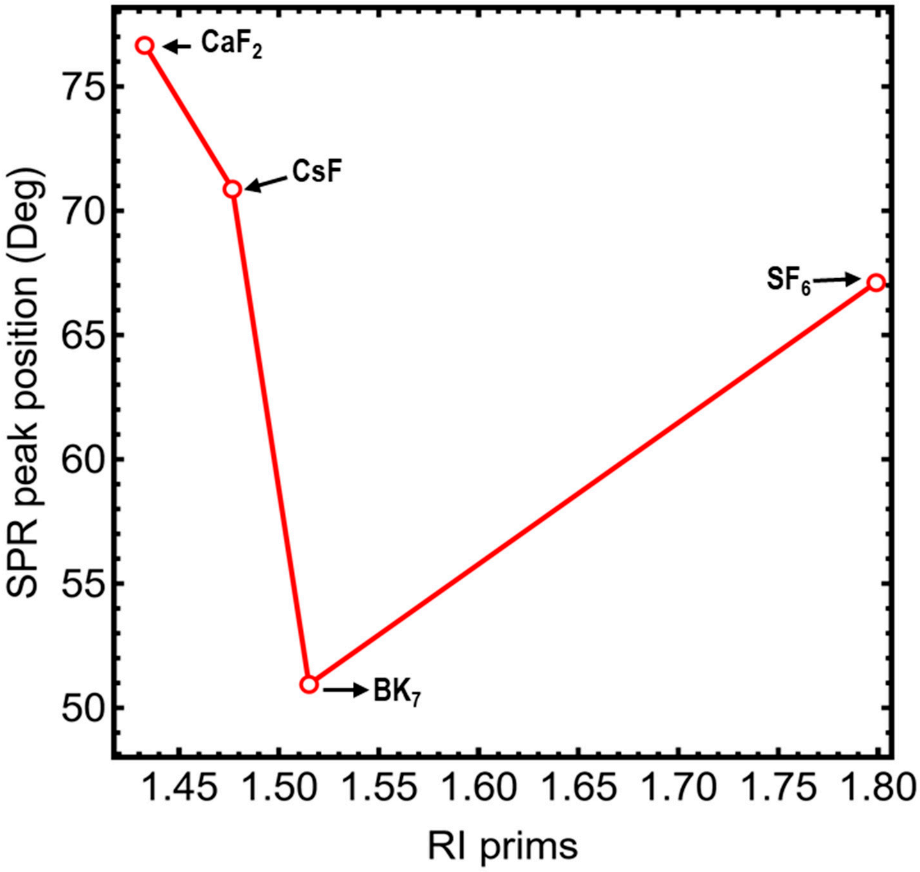

Figure S2. Relationship between SPR peak angle and refractive index of the prism.

Table S4. Refractive-index values for prism materials and corresponding SPR peak angles.

Code	RI Prism	SPR Peak Position
$Sys_{1\text{-}CaF2}$	1.4329	83.76
$Sys_{2\text{-}CsF}$	1.4768	75.48
$Sys_{3\text{-}SF6}$	1.7990	53.04
$Sys_{4\text{-}BK7}$	1.5151	70.95

Table S5. Description of simulated multilayer systems, including Au thickness and analyte conditions.

Code	Full Name	Short Name
$Sys_{0\text{-}Au}$	CaF_2/Gold/Water	CaF_2/Au/H_2O
$Sys_{1\text{-}Au\text{-}30nm}$	CaF_2/Gold $_{30nm}$/Carcinoma-$_{1ng/mL}$	CaF_2/Au$_{\text{-}30nm}$/Carcinoma$_{\text{-}1ng/mL}$
$Sys_{2\text{-}Au\text{-}35nm}$	CaF_2/Gold $_{35nm}$/Carcinoma-$_{1ng/mL}$	CaF_2/Au$_{\text{-}35nm}$/Carcinoma$_{\text{-}1ng/mL}$
$Sys_{3\text{-}Au\text{-}40nm}$	CaF_2/Gold $_{40nm}$/Carcinoma-$_{1ng/mL}$	CaF_2/Au$_{\text{-}40nm}$/Carcinoma$_{\text{-}1ng/mL}$
$Sys_{4\text{-}Au\text{-}45nm}$	CaF_2/Gold $_{45nm}$/Carcinoma-$_{1ng/mL}$	CaF_2/Au$_{\text{-}45nm}$/Carcinoma$_{\text{-}1ng/mL}$

Table S6. Optical performance metrics: resonance angle, Δθ, sensitivity, attenuation, and FWHM.

Code	SPR Peak Position	$\Delta\theta$ (Deg)	Sensitivity Enhancement (%)	Attenuation (%)	FWHM
$Sys_{1\text{-}Au\text{-}30nm}$	80.64	0.40	2.28	40.15	7.89
$Sys_{2\text{-}Au\text{-}35nm}$	81.73	0.19	0.96	19.01	6.95
$Sys_{3\text{-}Au\text{-}40nm}$	82.79	0.03	0.31	3.31	6.34
$Sys_{4\text{-}Au\text{-}45nm}$	83.75	0.01	1.48	1.03	6.03

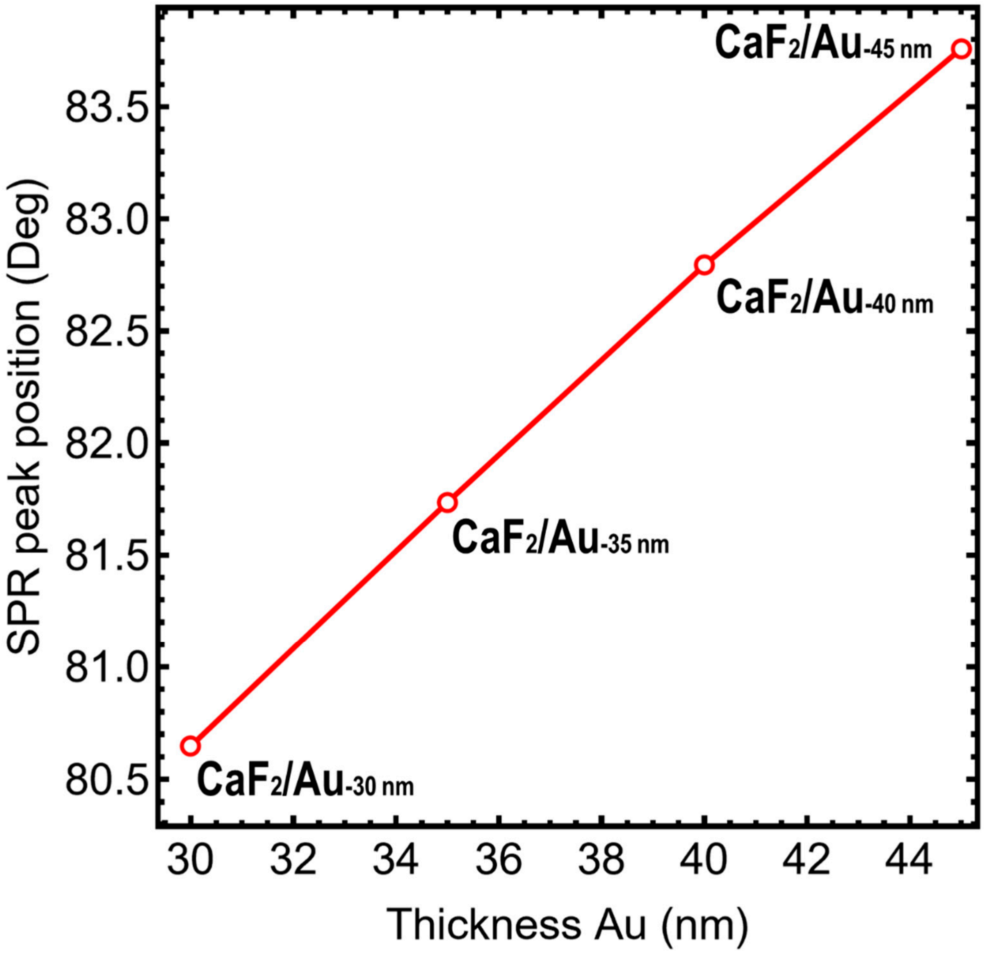

Figure S3. SPR resonance angle as a function of Au thickness.

Table S7. Refractive index (real part) of gold and corresponding SPR peak position for each configuration.

Code	RI Metal (Real Part)	SPR Peak Position
$Sys_{1\text{-}Au\text{-}30nm}$	0.1378	80.64
$Sys_{2\text{-}Au\text{-}35nm}$		81.73
$Sys_{3\text{-}Au\text{-}40nm}$		82.79
$Sys_{4\text{-}Au\text{-}45nm}$		83.75

Table S8. Descriptions of simulated multilayer systems with varying Si_3N_4 thickness and analyte conditions.

Code	Full Name	Short Name
$Sys_{0\text{-}Au+Si3N4}$	CaF_2/Gold/Silicone Nitride/Water	$CaF_2/Au/Si_3N_4/H_2O$
$Sys_{1\text{-}Au+Si3N4\text{-}2nm}$	CaF_2/Gold/Silicone Nitride$_{\text{-}2nm}$/Carcinoma$_{\text{-}1ng/mL}$	$CaF_2/Au/Si_3N_{4\text{-}2nm}$/Carcinoma$_{\text{-}1ng/mL}$
$Sys_{2\text{-}Au+Si3N4\text{-}3nm}$	CaF_2/Gold/Silicone Nitride$_{\text{-}3nm}$/Carcinoma$_{\text{-}1ng/mL}$	$CaF_2/Au/S_3N_{4\text{-}3nm}$/Carcinoma$_{\text{-}1ng/mL}$
$Sys_{3\text{-}Au+Si3N4\text{-}4nm}$	CaF_2/Gold/Silicone Nitride$_{\text{-}4nm}$/Carcinoma$_{\text{-}1ng/mL}$	$CaF_2/Au/Si_3N_{4\text{-}4nm}$/Carcinoma$_{\text{-}1ng/mL}$
$Sys_{4\text{-}Au+Si3N4\text{-}5nm}$	CaF_2/Gold/Silicone Nitride$_{\text{-}5nm}$/Carcinoma$_{\text{-}1ng/mL}$	$CaF_2/Au/Si_3N_{4\text{-}5nm}$/Carcinoma$_{\text{-}1ng/mL}$

Table S9. Key optical metrics: resonance position, Δθ, sensitivity, attenuation, and FWHM.

Code	SPR Peak Position	$\Delta\theta$ (Deg)	Sensitivity Enhancement (%)	Attenuation (%)	FWHM
$Sys_{1\text{-}Au+Si3N4\text{-}2nm}$	82.57	2.83	3.31	34.94	8.25
$Sys_{2\text{-}Au+Si3N4\text{-}3nm}$	83.74	1.66	1.95	30.62	8.18
$Sys_{3\text{-}Au+Si3N4\text{-}4nm}$	85.11	0.29	0.34	24.02	8.09
$Sys_{4\text{-}Au+Si3N4\text{-}5nm}$	86.73	1.32	1.55	13.31	8.02

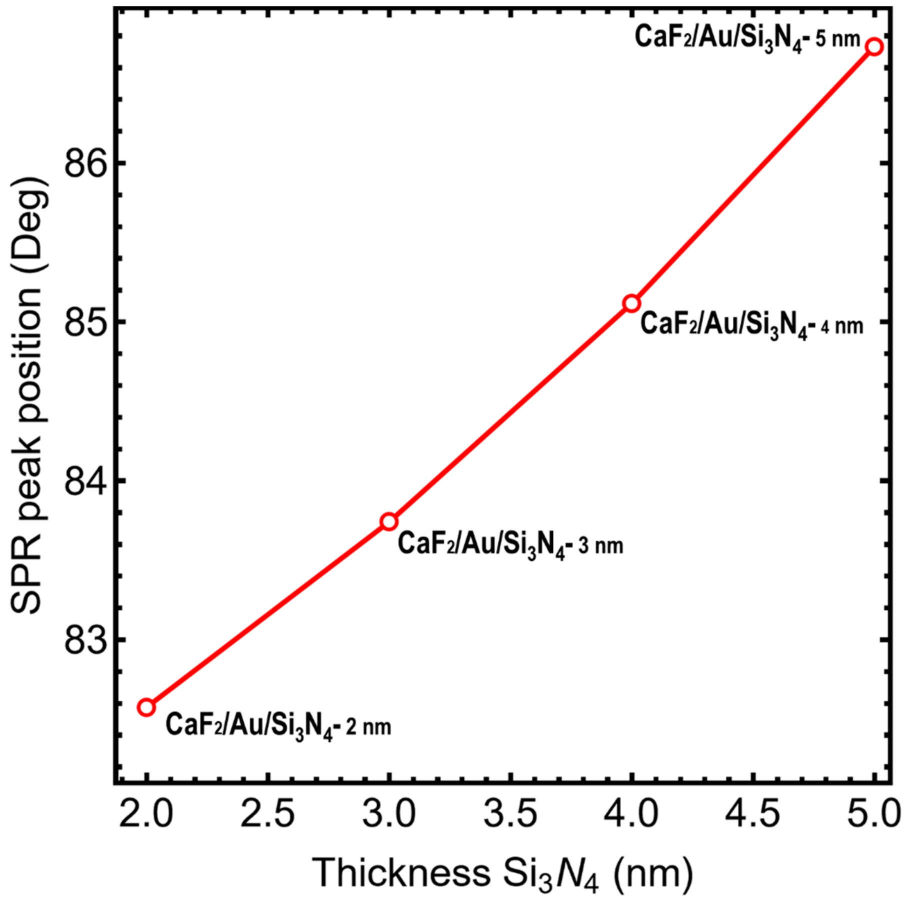

Figure S4. SPR resonance angle as a function of Si_3N_4 thickness.

Table S10. SPR peak angles and corresponding real part of the Si_3N_4 refractive index.

Code	RI Metal (Real Part)	SPR Peak Position
$Sys_{1\text{-}Au+Si3N4\text{-}2nm}$	2.0394	82.57
$Sys_{2\text{-}Au+Si3N4\text{-}3nm}$		83.74
$Sys_{3\text{-}Au+Si3N4\text{-}4nm}$		85.11
$Sys_{4\text{-}Au+Si3N4\text{-}5nm}$		86.73

Table S11. Descriptions of each system with corresponding nanomaterial and analyte conditions.

Code	Full Name	Short Name
$Sys_{0\text{-}CaF_2/Au/Si_3N_4/G/H_2O}$	CaF_2/Gold/Silicone Nitride/Graphene/Water	$CaF_2/Au/Si_3N_4/G/H_2O$
$Sys_{1\text{-}CaF_2/Au/Si_3N_4/BP/Carcinoma\text{-}1ng/mL}$	CaF_2/Gold/Silicone Nitride/Black Phosphorus/$Carcinoma_{\text{-}1ng/mL}$	$CaF_2/Au/Si_3N_4/BP/Carcinoma_{\text{-}1ng/mL}$
$Sys_{2\text{-}CaF_2/Au/Si_3N_4/MoS_2/Carcinoma\text{-}1ng/mL}$	CaF_2/Gold/Silicone Nitride/Molybdenum Disulfide/$Carcinoma_{\text{-}1ng/mL}$	$CaF_2/Au/Si_3N_4/MoS_2/Carcinoma_{\text{-}1ng/mL}$
$Sys_{3\text{-}CaF_2/Au/Si_3N_4/WS_2/Carcinoma\text{-}1ng/mL}$	CaF_2/Gold/Silicone Nitride/Tungsten Disulfide/$Carcinoma_{\text{-}1ng/mL}$	$CaF_2/Au/Si_3N_4/WS_2/Carcinoma_{\text{-}1ng/mL}$
$Sys_{4\text{-}CaF_2/Au/Si_3N_4/G/Carcinoma\text{-}1ng/mL}$	CaF_2/Gold/Silicone Nitride/Graphene/$Carcinoma_{\text{-}1ng/mL}$	$CaF_2/Au/Si_3N_4/G/Carcinoma_{\text{-}1ng/mL}$

Table S12. SPR metrics: peak position, Δθ, sensitivity, attenuation, and FWHM for each nanomaterial.

Code	SPR Peak Position	$\Delta\theta$ (Deg)	Sensitivity Enhancement (%)	Attenuation (%)	FWHM
$Sys_{1\text{-}CaF_2/Au/Si_3N_4/BP/Carcinoma\text{-}1ng/mL}$	86.97	2.29	2.71	11.15	7.94
$Sys_{2\text{-}CaF_2/Au/Si_3N_4/MoS_2/Carcinoma\text{-}1ng/mL}$	87.23	2.55	3.01	2.28	9.18
$Sys_{3\text{-}CaF_2/Au/Si_3N_4/WS_2/Carcinoma\text{-}1ng/mL}$	88.02	3.34	3.94	7.98	8.65
$Sys_{4\text{-}CaF_2/Au/Si_3N_4/G/Carcinoma\text{-}1ng/mL}$	85.88	1.20	1.41	10.61	8.33

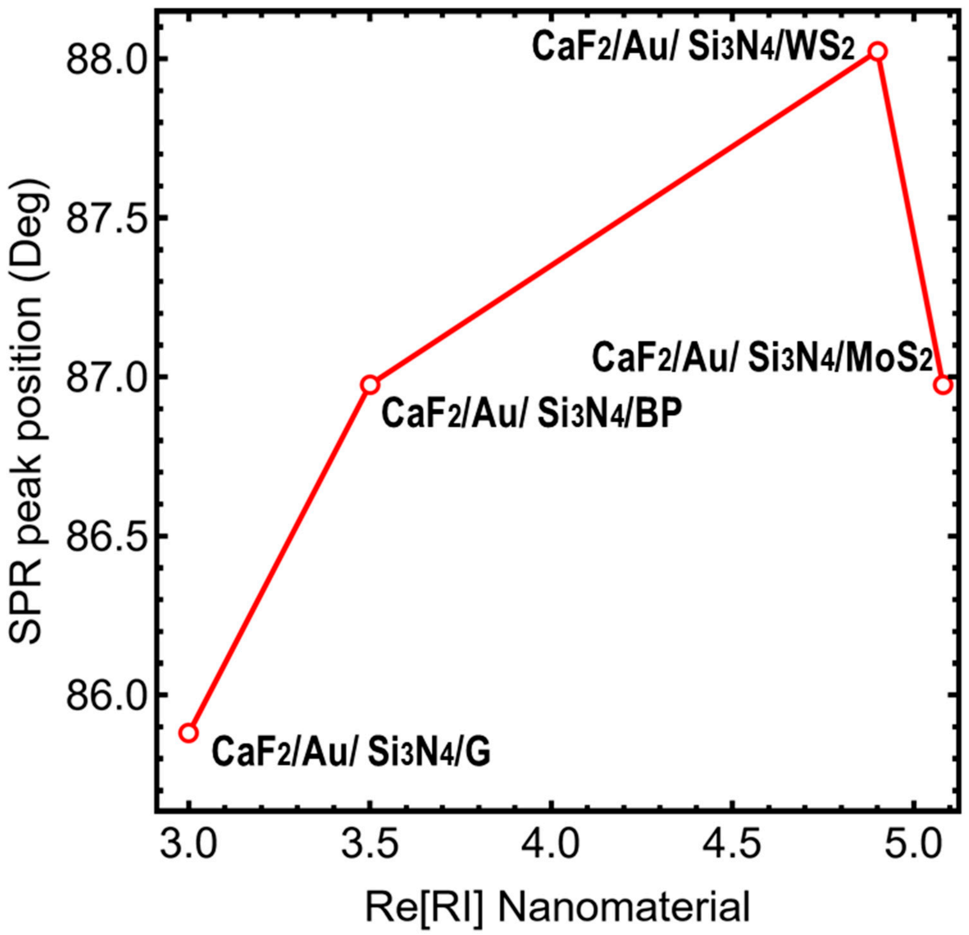

Figure S5. SPR resonance angle as a function of nanomaterial thickness.

Table S13. Refractive-index properties of nanomaterials and resulting SPR peak angles.

Code	RI Nanomaterial	Im	SPR Peak Position
Sys1-CaF_2/Au/Si_3N_4/BP/Carcinoma-1ng/mL	3.5000	0.0100	86.97
Sys2-CaF_2/Au/Si_3N_4/MoS_2/Carcinoma-1ng/mL	5.0805	1.1723	87.23
Sys3-CaF_2/Au/Si_3N_4/WS_2/Carcinoma-1ng/mL	4.9000	0.3124	88.02
Sys4-CaF_2/Au/Si_3N_4/G/Carcinoma-1ng/mL	3.0000	1.1491	85.88

Table S14. Refractive index (RI) values corresponding to different carcinoma concentrations (1–5 ng/mL) used to simulate biological variation in the sensing environment.

Carcinoma (ng/mL)	RI
1	1.3337
2	1.3374
3	1.3411
4	1.3448
5	1.3485

Table S15. Optical constants and layer thicknesses used in the optimized $CaF_2/Au/Si_3N_4/BP$ Kretschmann SPR configuration, including the baseline aqueous medium and the effective-refractive-index values used to represent biomarker-binding conditions (1–5 ng/mL).

Material	Refractive Index	Thickness (nm)
CaF_2	1.4329	---
Gold (Au)	0.1378 + 3.6196i	30.0
Silicone Nitride (Si_3N_4)	2.0394	4.0
Black Phosphorus (BP)	3.5 + 0.01i	0.53
Deionized Water (DIW)	1.331700	---
Carcinoma -1ng/mL	1.333700	---
Carcinoma -2ng/mL	1.337400	---
Carcinoma -3ng/mL	1.341100	---
Carcinoma -4ng/mL	1.344800	---
Carcinoma -5ng/mL	1.348500	---

Table S16. Performance metrics of the optimized $CaF_2/Au/Si_3N_4/BP$ SPR biosensor under baseline water and effective binding conditions (1–5 ng/mL): SPR peak position, angular shift ($\Delta\theta$), sensitivity enhancement, attenuation, and FWHM.

Code	SPR Peak Position	$\Delta\theta$ (Deg)	Sensitivity Enhancement (%)	Attenuation (%)	FWHM
$Sys_{1\text{-}CaF_2/Au/Si_3N_4/BP/DIW}$	85.67	0.82	0.96	2.59	3.87
$Sys_{2\text{-}CaF_2/Au/Si_3N_4/BP/1\ ng/mL}$	86.76	1.91	2.25	9.39	4.13
$Sys_{3\text{-}CaF_2/Au/Si_3N_4/BP/2\ ng/mL}$	87.71	2.86	3.37	56.24	5.02
$Sys_{4\text{-}CaF_2/Au/Si_3N_4/BP/3\ ng/mL}$	87.04	2.19	2.58	84.42	7.01
$Sys_{5\text{-}CaF_2/Au/Si_3N_4/BP/4\ ng/mL}$	86.39	1.54	1.81	92.39	6.55
$Sys_{6\text{-}CaF_2/Au/Si_3N_4/BP/5\ ng/mL}$	66.00	18.85	22.21	93.99	21.47

Table S17. Effective-refractive-index values used to represent biomarker-binding conditions (1–5 ng/mL) and the corresponding SPR resonance-angle positions of the $CaF_2/Au/Si_3N_4/BP$ sensor.

Code	RI	SPR Peak Position
$Sys_{1\text{-}CaF_2/Au/Si_3N_4/BP/DIW}$	1.331700	85.67
$Sys_{2\text{-}CaF_2/Au/Si_3N_4/BP/1\ ng/mL}$	1.333700	86.76
$Sys_{3\text{-}CaF_2/Au/Si_3N_4/BP/2\ ng/mL}$	1.337400	87.71
$Sys_{4\text{-}CaF_2/Au/Si_3N_4/BP/3\ ng/mL}$	1.341100	87.04
$Sys_{5\text{-}CaF_2/Au/Si_3N_4/BP/4\ ng/mL}$	1.344800	86.39
$Sys_{6\text{-}CaF_2/Au/Si_3N_4/BP/5\ ng/mL}$	1.348500	66.00

Table S18. Sensitivity, detection accuracy, and quality-factor values for each biosensor system as a function of modeled carcinoma concentrations.

Modeled Concentrations	Modeled RI	S (°/RIU)	DA	QF (RIU^{-1})
Carcinoma 1 ng/mL	1.333700	482.82	0.211	124.60
Carcinoma 2 ng/mL	1.337400	517.62	0.463	125.28
Carcinoma 3 ng/mL	1.341100	386.59	0.569	76.90

Carcinoma 4 ng/mL	1.344800	197.62	0.312	28.15
Carcinoma 5 ng/mL	1.348500	104.10	0.235	15.88

Table S19. Quantitative values of figure of merit, limit of detection, and contrast signal factor derived from modeled RI shifts for carcinoma concentrations between 1 and 5 ng/mL.

Modeled Concentrations	Modeled RI	FoM (RIU^{-1})	LoD (10^{-5})	CSF
Carcinoma 1 ng/mL	1.333700	199.13	1.03	205.84
Carcinoma 2 ng/mL	1.337400	1051.52	0.96	1058.40
Carcinoma 3 ng/mL	1.341100	4248.23	1.29	4252.58
Carcinoma 4 ng/mL	1.344800	2348.46	2.53	2350.09
Carcinoma 5 ng/mL	1.348500	1452.19	4.80	1453.13
